# Comprehensive Analysis of Advancement in Optical Biosensing Techniques for Early Detection of Cancerous Cells

**DOI:** 10.3390/bios15050292

**Published:** 2025-05-05

**Authors:** Ayushman Ramola, Amit Kumar Shakya, Arik Bergman

**Affiliations:** Department of Electrical and Electronics Engineering, Ariel University, Ariel 40700, Israel; ayushmanr@ariel.ac.il (A.R.); arikb@ariel.ac.il (A.B.)

**Keywords:** *SPR* sensors, *LSPR* sensors, colorimetric sensors, fluorescence-based sensors, photonics and waveguide sensors, fiber optic sensors, *SERS*

## Abstract

This investigation presents an overview of various optical biosensors utilized for the detection of cancer cells. It covers a comprehensive range of technologies, including surface plasmon resonance (SPR) sensors, which exploit changes in refractive index (RI) at the sensor surface to detect biomolecular interactions. Localized surface plasmon resonance (LSPR) sensors offer high sensitivity and versatility in detecting cancer biomarkers. Colorimetric sensors, based on color changes induced via specific biochemical reactions, provide a cost-effective and simple approach to cancer detection. Sensors based on fluorescence work using the light emitted from fluorescent molecules detect cancer-specific targets with specificity and high sensitivity. Photonics and waveguide sensors utilize optical waveguides to detect changes in light propagation, offering real-time and label-free detection of cancer biomarkers. Raman spectroscopy-based sensors utilize surface-enhanced Raman scattering (SERS) to provide molecular fingerprint information for cancer diagnosis. Lastly, fiber optic sensors offer flexibility and miniaturization, making them suitable for in vivo and point-of-care applications in cancer detection. This study provides insights into the principles, applications, and advancements of these optical biosensors in cancer diagnostics, highlighting their potential in improving early detection and patient outcomes.

## 1. Introduction

Cancer is among the most fatal diseases infecting human beings of the 21st century. The modern understanding of cancer began to develop in the 19th century with pivotal advancements in medical science, including the development of cell theory and innovations in anesthesia and antiseptic techniques that revolutionized surgery. The discovery of X-rays in 1895 and the development of radiation therapy, along with the emergence of chemotherapy in the 1940s, marked early milestones in non-surgical cancer treatments. The latter half of the 20th century was defined by breakthroughs in molecular biology, most notably the discovery of DNA’s structure in 1953, which paved the way for identifying genetic mutations responsible for cancer [1,2,3]. This era also saw the advent of hormone therapy, immunotherapy, and the concept of targeted therapy, proceeding to complete the human genome project by 2003, which heralded the age of precision medicine offering treatments tailored to the genetic profile of individual cancers [4]. Despite all of these advancements, cancer remains one of the leading causes of disease and death worldwide, with early and accurate detection playing a critical role in improving patient outcomes. Traditional diagnostic methods such as imaging and biopsy are effective but often suffer from limitations including invasiveness, high costs, and delayed results. In recent years, the demand for rapid, sensitive, and non-invasive diagnostic technologies has attracted significant research into biosensing approaches. Among these, optical biosensors have gained a lot of popularity due to their ability to detect cancer-related biomarkers or circulating tumor cells (CTCs) with high sensitivity and specificity [5].

The 21st century has been characterized by significant advances in immunotherapy, such as checkpoint inhibitors and CART cell therapy, alongside ongoing research aimed at understanding cancer’s molecular underpinnings, improving treatment, reducing toxicity, and preventing cancer through lifestyle interventions and vaccines. This rapid evolution of cancer understanding and treatment over the past two centuries reflects a transition from rudimentary knowledge to a sophisticated, genetics-based approach that continues to offer new hope for effective cancer management and a cure [6,7,8]. However, when these regulatory processes fail, cells can begin to grow uncontrollably, leading to the formation of tumors or malignancies. Not all tumors are cancerous; for instance, benign tumors do not spread to other body parts. Malignant tumors, also known as cancerous tumors, can infiltrate surrounding tissues and disseminate into different parts of the body through the bloodstream, lymphatic system, cells, etc.; this process is referred to as metastasis. The precise origins of cancer typically involve a combination of factors, including genetic mutations, environmental influences, lifestyle decisions, e.g., smoking, dietary habits, etc., and contact with specific chemicals or radiation [9,10].

**Figure 1:**
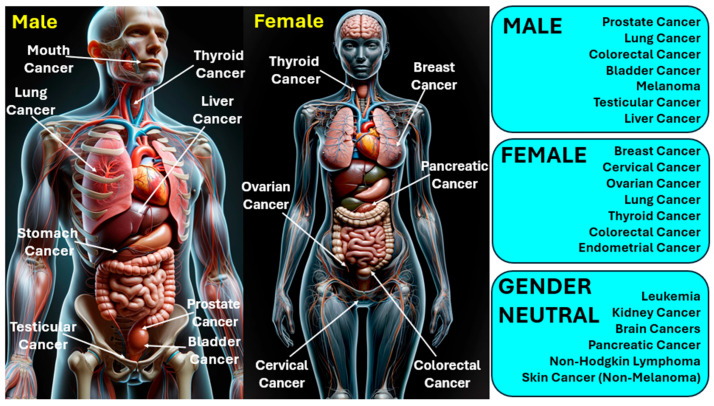
Classification of various types of cancers infecting human beings.

The management and outlook of cancer differ significantly based on its variety, stage, and other personal considerations. Treatment options may encompass surgical intervention, radiation therapy, chemotherapy, immunotherapy, and therapies that target specific aspects of cancer cells [11,12]. In 2020, cancer was a leading cause of mortality worldwide, claiming more than 10 million lives [13]. Cancer has various types; lung and breast cancers are notably prevalent across both genders. The most frequently identified cancers include carcinoma, lymphoma, leukemia, sarcoma, and melanoma, each capable of originating in different human organs [14]. Figure 1 presents the classification of various types of cancers infecting humans. Gender-neutral cancers are considered those cancer types that can affect both males and females. Factors such as radiation, over-aging, viral infections, prolonged exposure to the sun, smoking, hormone therapies, and exposure to certain chemicals are well-recognized contributors to cancer development [15].

Common methods for diagnosing cancer include mammography [16], biopsies of tissue [17], magnetic resonance imaging (MRI) [18], enzyme-linked immunosorbent assays (ELISA) [19], polymerase chain reaction (PCR) [20], sonography [21], and breast imaging based on molecules [22]. However, tissue biopsy, a standard diagnostic procedure, is not without its risks and is impractical for certain locations within the body [23]. Monitoring cancer progression and obtaining samples often necessitate surgical interventions. The typical protocols for detecting cancer face numerous challenges, including the need for skilled professionals, the requirement of large volumes of bodily fluids, expensive, complex equipment, and time-consuming processes [24]. Additionally, these diagnostic methods need millions of cells for an accurate diagnosis, which hampers early detection efforts. In response, researchers are working to create diagnostics that are more sensitive, quicker, and easier to use, aiming to overcome these limitations. Table 1 presents a summary of information on distinct methodologies that are used to detect a particular form of cancer in human beings.

Optical biosensors play a crucial role in cancer diagnostics by enabling the detection of specific biomarkers, deoxyribonucleic acid (DNA), ribonucleic acid (RNA), proteins, or whole tumor cells present in body fluids such as blood, saliva, cell fluid, or urine [45]. These sensors operate based on the interaction of light with biological elements, offering real-time monitoring, high sensitivity, and label-free detection capabilities. Optical sensing technologies have been extensively employed to recognize cancer-associated analytes at very low concentrations. Their ability to provide quantitative and qualitative information with minimal sample preparation makes them highly suitable for early-stage cancer detection [46].

Recent advancements in biosensing technology have focused on improving sensitivity, selectivity, and portability. Innovations in nanomaterials such as gold nanoparticles (AuNPs), graphene, and quantum dots have significantly improved signal amplification and detection accuracy for optical biosensors. Miniaturization through microfluidic integration has enabled point-of-care testing (POCT) with minimal sample volumes. Additionally, combining optical sensing platforms with data analytics and machine learning has improved interpretation and real-time decision-making [47,48]. These developments have made optical biosensors more reliable and applicable in early cancer diagnostics and continuous health monitoring.

**Figure 2:**
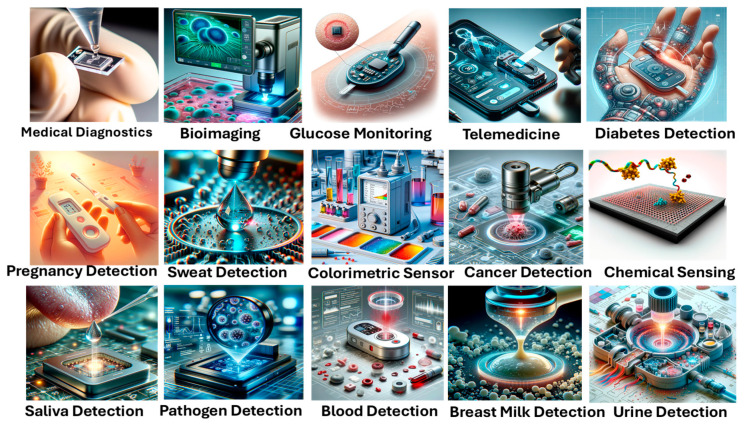
Medical applications of optical biosensors.

Optical biosensors have tremendous potential to be used for several applications related to health monitoring and early detection. In cancer detection and identification, they have also proven to be used as important medical devices. These sensors can be used in various other application areas related to food quality, environmental analysis, industrial applications, military applications, etc. However, this work focuses exclusively on their applications in cancer detection. Optical biosensors offer a non-invasive alternative to current cancer diagnosis methods. These devices utilize a biomarker to identify the target molecule, a bio-receptor for recognition, and a physicochemical transducer to detect the interaction. Optical biosensors typically rely on cancer-related biomaterials like microRNAs, proteins, circulating tumor cells, DNA, and exosomes (EXOs), which can be derived from human body fluids [49,50,51,52]. Figure 2 presents various medical applications of optical biosensors by using human body fluids as a noninvasive investigating analyte for disease identification.

Several optical biosensor-based setups have already been translated into practical applications for early cancer detection to date. Some of the prominent one include the Biacore SPR platform, which is widely used for biomolecular interaction analysis, including cancer biomarker detection in pharmaceutical and clinical research [53]. Veristrat system, a serum-based proteomic test, utilizes mass spectrometry and optical sensing for non-small cells related to lung cancer classification and treatment guidance [54]. Oncocyte’s DetermaDx are emerging optical biosensor kits designed for liquid biopsy approaches, detecting circulating tumor-derived nucleic acids from blood samples [55]. These platforms demonstrate the growing role of optical biosensing in non-invasive, rapid, and accurate cancer diagnostics.

This study presents an exhaustive analysis of various optical sensing techniques, optical sensors, specific biomarkers, binding materials, plasmonic materials, etc., used to detect and identify cancerous cells in the human body. In addition to this, the article presents details regarding various types of photonic crystal fiber (PCF), fiber Bragg gratings, waveguide structures, and resonance-based sensing methods such as localized surface plasmon resonance (LSPR) and surface plasmon resonance (SPR) specifically for cancer cell detection and early prediction.

## 2. Classification of Optical Biosensors

Optical biosensors have revolutionized the field of detection and analysis, offering a range of techniques suited for various applications. One of the prominent applications includes the identification and detection of cancerous cells. Among the diverse types of optical biosensors SPR sensors detect alteration in the refractive index (RI) near a sensor’s surface, making them invaluable in biomolecular interaction analysis [56,57,58,59]. Similarly, LSPR based sensors analyze the localized oscillations of conduction electrons at the nanoscale, offering enhanced sensitivity for detecting molecular changes [60,61,62]. Colorimetric sensors, which change color in response to analyte interaction, offer a simple and direct method of visual detection, making them highly accessible for various applications [63,64,65]. Fluorescence-based sensors are distinguished by their ability to emit light upon excitation, providing high specificity and sensitivity for targeted molecule detection [66,67,68]. Photonics and waveguide sensors utilize the principles of light guidance and manipulation to detect changes in light properties or interaction with materials, offering a platform for high-throughput screening [69,70,71]. Finally, Raman spectroscopy-based sensors leverage the Raman scattering effect to provide detailed information about molecular vibrations, enabling precise chemical identification [72,73,74]. Interferometric sensors, employing the principle of optical interference, offer ultrasensitive detection capabilities, making them ideal for measuring minute changes in biological samples. Fiber-optic sensors, known for their flexibility and remote sensing capabilities, utilize light modulation within optical fibers (OF) to detect changes in the external environment, offering a robust solution for in-situ monitoring [75,76,77]. Lastly, together, these optical sensors provide a comprehensive toolkit for advanced detection and analysis in various scientific and medical fields. Thus, this study presents details regarding the procedure to use body fluid liquid as analyte, preparation for biomarker testing, molecular testing, etc., that can be merged with optical sensors for cancer detection and identification.

Types of optical sensors are listed as follows.

- SPR sensors [39,40,41].
- LSPR sensors [60,62].
- Colorimetric sensors [63,64,65].
- Fluorescence-based sensors [66,67,68].
- Photonics and waveguide sensors [69,70,71].
- Raman spectroscopy-based sensors [72,73,74].
- Fiber optic sensors [75,76,77].

## 3. Advanced Optical Biosensors Technologies and Applications

### 3.1. Classification of SPR Sensors

**Figure 3:**
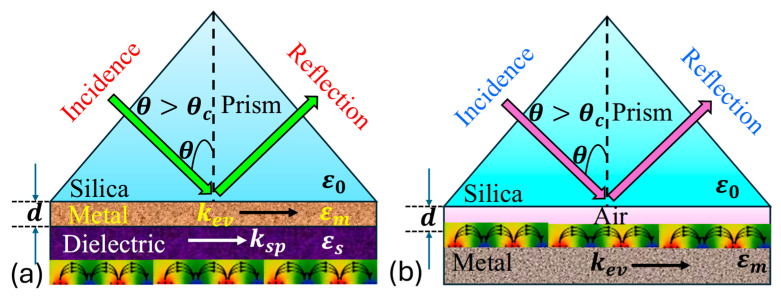
Illustration of (**a**) *Kretschmann* configuration [78], (**b**) *Otto* configuration [78].

SPR involves the interaction between a surface plasmon wave (SPW) and electromagnetic wave, also referred to as a surface plasmon polariton (SPP), occurring at the boundary between a metal and a dielectric medium. The SPW is characterized as a transverse magnetic (TM) polarized electromagnetic wave, exhibits evanescent decay into adjacent media. Its magnetic vector stands perpendicular to the propagation direction while remaining parallel to the plane of the interface. SPR occurs when there is a resonance between the frequencies and parallel components of an incident TM polarized electromagnetic wave and the SPW. This phenomenon is observed in attenuated total reflection setups within a prism-coupling configuration. SPR enables the energy transfer from incoming photons to surface plasmons (SPs), resulting in a reduction in the energy in the reflected light. Initially observed on the *Otto* configuration with a silver (Ag) film by *Otto*, it was later refined by *Kretschmann*. The *Kretschmann* and *Otto* configuration exemplifies the principles underlying SPR, as presented in Figure 3 [78].

Based on Maxwell’s equations and associated boundary conditions, the propagation constant $k_{sp}$ for an SPW traversing the boundary between a metal and a semi-infinite dielectric material can be determined via Equation (1). Here, $\varepsilon_{s}$ represents the dielectric constant of the dielectric material, $\varepsilon_{m}\;$denotes the dielectric constant of the metal, ω is the frequency of the incident wave, and c stands for the speed of light [79].

(1) $$k_{sp}=\frac{\omega }{c}\sqrt{\frac{\varepsilon_{m}\varepsilon_{s}}{\varepsilon_{m}+\varepsilon_{s}}}$$

In the context of the *Kretschmann* configuration, the propagation constant of the evanescent field, which is parallel to the metal surface, is detailed in Equation (2) [79].

(2) $$k_{ev}=\frac{\omega }{c}\sqrt{\varepsilon_{0}}\;sin\theta$$

The SPR phenomenon occurs when the frequency of the evanescent field matches the SPW frequency. This is expressed in Equation (3), where $\theta_{res}$ is called the resonance angle [79].

(3) $$\frac{\omega }{c}\;sin\theta_{res}=\frac{\omega }{c}\sqrt{\frac{\varepsilon_{m}\varepsilon_{s}}{{\varepsilon_{0}(\varepsilon }_{m}+\varepsilon_{s})}}$$

Thus, in the *Kretschmann* configuration, a metal film is deposited onto a dielectric substrate, which is typically a glass prism. This setup allows light to hit at an angle above the critical threshold for total internal reflection (TIR), creating an evanescent wave in the metal film that couples with SPs at the metal–dielectric boundary. Contrastingly, the *Otto* configuration involves sandwiching a metal film between two dielectric layers, with incident light typically directed onto the upper dielectric layer. Both configurations facilitate SPR observation, but the *Kretschmann* setup is more relevant due to its higher sensitivity and widespread application in biosensing, while the *Otto* configuration offers advantages in certain scenarios, such as simpler fabrication processes and reduced sensitivity to changes in the surrounding medium’s RI.

Another type of SPR-based sensor is designed using PCF. They are also known as micro-structured or holey fibers. There are types of OF having a periodic array of air holes along the length of the fiber. Unlike traditional OF, which rely on TIR, PCFs guide light via structural dispersion through their micro-structured cross-section [80,81]. This unique construction allows for the tailoring of optical properties, such as dispersion, nonlinearity, and the guiding of light in air or vacuum-filled cores. SPR occurs when light triggers the collective oscillation of electrons at the boundary between materials with negative and positive permittivity, typically at a metal-dielectric boundary.

**Figure 4:**
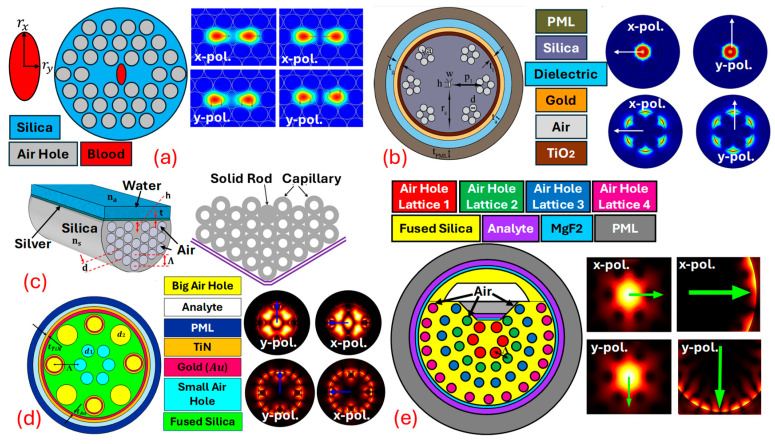
Types of PCF SPR sensor models. (**a**) IMD-shaped PCF [87]. (**b**) EMD-shaped PCF [88]. (**c**) D-shaped PCF [89]. (**d**) Merger of IMD and EMD PCF [86]. (**e**) Fusion of quasi-D-shaped and EMD PCF [59].

This resonance condition is highly sensitive to changes in the RI near the metal surface, making SPR an effective sensing mechanism for detecting chemical and biological analytes [82,83]. Combining PCFs with SPR leads to highly sensitive and selective sensors. PCF-based SPR sensors utilize the unique guiding properties of PCF to expose the evanescent field of light propagating within the fiber to an external medium. By coating parts of the PCF with a thin layer of plasmonic metals, the evanescent field can interact with SPs on the metal surface. This interaction is sensitive to changes in the RI of the surrounding medium, which can be used to detect various analytes with high precision. PCF SPR sensors can be designed using several geometries, but the main challenge is the real-time fabrication of the fiber. As for PCF fabrication, high-end equipment and machinery are required [84,85]. Thus, currently, the PCF SPR sensors are mostly limited to theoretical design and analysis. PCF SPR sensors are designed using three design procedures, which include internal metal deposition (IMD), external metal deposition (EMD), and the D-shaped designing method; in addition to these traditional approaches, PCF can also be fabricated using the slotted-shaped approach [86]. In IMD-shaped PCF SPR sensors’ liquid analyte is passed from inside the PCF. Different air-hole geometries are possible, which assists in the interaction of the analyte with the PCF. Figure 4 represents a variety of PCF SPR sensor models based on different design approaches. Figure 4a represents the IMD-shaped PCF SPR sensor with x-pol. and y-pol. being polarized core modes [87]. Figure 4b represents a PCF SPR sensor model based on the EMD design methodology. In EMD-shaped PCF SPR sensors, the analyte channel is present at the exterior surface of the fiber body, having coats of plasmonic materials that assist in the generation of the SPP at the metal–dielectric interface [88]. Figure 4c represents the D-shaped PCF SPR sensor model. D-shaped PCF SPR sensor models possess a coat of plasmonic materials on a polished flat surface. The polished surface needs to be designed with extreme care as, during polishing, there is always a possibility of disturbing the air-hole geometry [89]. In recent times, researchers have come up with a novel model of PCF SPR sensors in which they merge two different design techniques to develop a new sensor design methodology. In this quest, Figure 4d represents a PCF SPR sensor model that achieves a merger of the IMD and EMD design methodology in the single PCF SPR sensor for transformer oil sensing [86]. Similarly, Figure 4e represents a novel design of the PCF SPR sensor model with a merger of quasi-D-shaped PCF and an EMD technique for heavy-metal sensing [59].

As suggested above, a variety of plasmonic materials are used in the sensor models, which include gold (Au), Ag, aluminum (Al), indium tin oxide (ITO), titanium dioxide $\left(TiO_{2}\right)$, graphene, etc. [82]. Innovation and exploration are also performed to identify various substitutes for expensive plasmonic materials, and researchers have identified materials like transition-metal dichalcogenides (TMDCs) [90], transparent conductive oxides (TCOs) [91], magnesium fluoride $(MgF_{2})\;$[92], MXenes [93], perovskites [94], silicene [91], phosphorene [91], etc. Infiltrating the PCF SPR surface can be achieved through various methods, such as sputtering, evaporation, or electroless plating [95]. A further analyte is infiltrated in the PCF surface to enhance the interaction between the guided light and the analyte in order to achieve these objective methods; for instance, precision injection and capillary forces are used. Plasmonic materials can be deposited over the PCF using a number of techniques, among which chemical vapor deposition (CVD) is considered the most prominent [96]. Several imaging techniques can be used to obtain a sophisticated image of the designed PCF when fabricated. These techniques include transmission electron microscopy (TEM) [97], scanning electron microscopy (SEM) [98], atomic force microscopy (AFM) [99], optical microscopy [100], etc.

**Figure 5:**
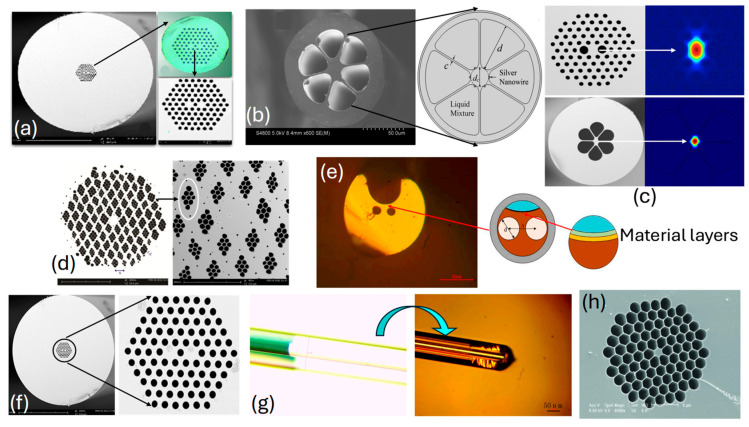
SEM images of the fabricated PCF (**a**) hexagonal lattice of air holes [101], (**b**) lattice with six air holes [104], (**c**) hexagonal lattice of air holes [105], (**d**) hexagonal lattice of parallelogram-shaped air holes [103], (**e**) SEM image-slotted PCF [106], (**f**) hexagonal lattice of air holes with dual-core configuration [102], (**g**) SEM image of ultrathin PCF [106], and (**h**) air holes in hexagonal honeycomb PCF [107].

The real-time fabricated PCF with different design configurations is presented in Figure 5. Figure 5a,d,f represent fabricated PCF with a hexagonal lattice of air-hole geometry; see [101,102,103], respectively. Figure 5b represents fabricated PCF with six air holes, and Figure 5c represents fabricated PCF with a hexagonal lattice of air holes, [104,105], respectively. Figure 5e represents an SEM image of a slotted PCF [106]. Figure 5g represents an SEM image of a fabricated fiber with an ultrathin core [106], finally, Figure 5h represents an SEM image of a fabricated hexagonal honeycomb PCF model [107]. Thus, PCF SPR sensor models presented in the literature for different applications can be fabricated, but only with the assistance of high-end machinery and equipment.

**Figure 6:**
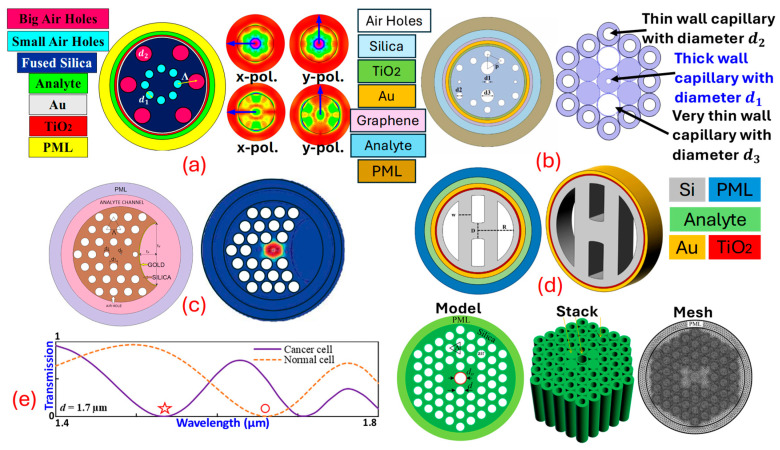
PCF SPR sensor models dedicated to cancer detection. (**a**) EMD-shaped PCF [6]. (**b**) Dual-core EMD-shaped PCF [108]. (**c**) Slotted IMD-shaped PCF [109]. (**d**) H-shaped PCF [110]. (**e**) Quad-core configuration with hexagonal-lattice PCF [111].

PCF SPR sensors can be used significantly for cancer detection. *Ramola* et al. [6], in Figure 6a, represent the PCF SPR sensor based on the EMD approach. Cancerous cells named squamous cells, basal cells, HeLa, INBL, CaSki (HIC), Jurkat, JM (JJM), PC12, MDA-MB-231 (MM231), and MCF7 related to skin, cervical, blood, adrenal gland, and breast cancer (Types 1 and 2), are identified from their proposed sensor. A dual-mode investigation corresponding to TM polarization (x-pol.) and transverse electric (TE) polarization (y-pol.) is performed. A high wavelength sensitivity (WS) of 12,857.14 nm RIU^−1^, an amplitude sensitivity (AS) of 15,010 RIU^−1^, and a sensor resolution (SR) in the order of 10^−6^ is obtained from the proposed biosensor. *Ibrahimi* et al. [108] in Figure 6b present a dual-core PCF SPR biosensor with a triple coat of Au/$TiO_{2}$/graphene as a plasmonic material. Cervical, blood, adrenal gland, and breast cancer (Types 1 and 2) are detected from the proposed biosensor. The highest WS of $5714.29\;\mathrm{nm}\;{RIU}^{-1}$, AS of $599.33\;{RIU}^{-1}$, and SR in the order of $10^{-5}\;RIU$ are obtained from their proposed biosensor. *Yasli* [109], in Figure 6c, presents a novel model of a PCF SPR sensor for cancer cell detection with a slot inside the background material of the PCF. The biosensor model has a coat of Au for SPW generation. The biosensor is built using the finite element method (FEM)-based approach. Six different cancers have been detected using their sensor model. The highest WS of 7142.86 nm/RIU and AS of $757\;{RIU}^{-1}$ is obtained from the biosensor model. *Pappu* et al. [110], in Figure 6d, present an Au-coated, H-shaped biosensor model for cancer cell identification. The proposed biosensor investigates skin cancer, cervical cancer, and breast cancer cells. The maximum WS obtained from the proposed biosensor is $7857.14\;\mathrm{nm}\;{RIU}^{-1}$, with an AS of $985.26\;{RIU}^{-1}\;$and an SR in the order of $10^{-5}\;RIU$, respectively. *Mollah* et al. [111], in Figure 6e, present a PCF SPR sensor based on a hexagonal lattice of air holes for blood cancer detection. The stacked preform of the proposed sensor model is also presented. They obtained a WS of 8571.43 nm/RIU from their designed sensor model. Similarly, several PCF SPR sensor models can be designed based on different design approaches and can be used effectively for cancer detection. In addition to these popular *SPR* techniques, the *SPR* phenomenon is also used in the following sensor technologies.

Waveguide-based *SPR* sensors: These integrate waveguides with *SPR* techniques, through which plasmons are excited along the metal-coated waveguide. They are compact and useful for integrated lab-on-a-chip systems [112,113,114,115].

Grating-based *SPR* sensors use a diffraction grating instead of a prism to excite SPs. These are advantageous for their simplicity, possess potential for large-scale development, and offer cost-effective production [116,117,118,119].

Photodetector-based *SPR* sensors combine *SPR* technology with photodetectors to measure the intensity of plasmonic light directly, offering compact solutions for portable sensing systems [120,121,122].

*SPR* imaging (*SPRI*): an advanced technique through which the reflected *SPR* signal is captured as an image, enabling high-throughput sensing and an analysis of multiple samples simultaneously [123,124,125,126].

### 3.2. Classification of LSPR Sensors

**Figure 7:**
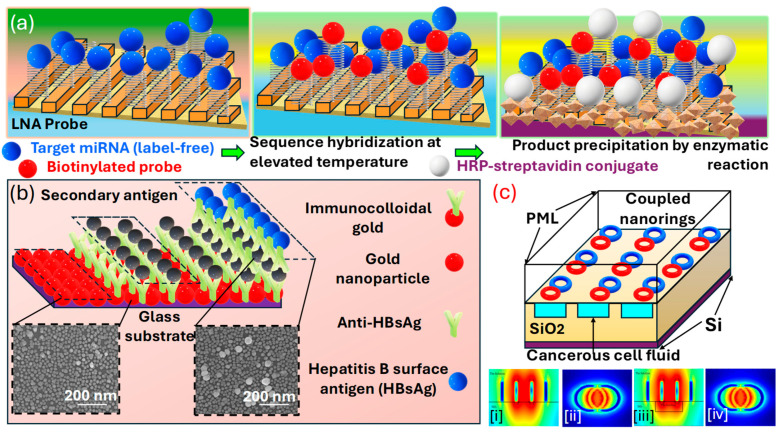
(**a**) Illustration of the enhanced signal LSPR miRNA detection system using a scalable, flexible, and transparent 3D plasmonic nanoarchitecture. The formation of a SAM incorporating a hairpin LNA probe, hybridization with miRNAs at an increased temperature, followed by the application of a biotin-tagged signaling probe, and finally interaction with HRP-conjugated streptavidin leading to an enzymatic conversion of a soluble substrate into an insoluble deposit; detecting HBsAg using a one-step LSPR sensor chip configuration, along with an improved LSPR chip design for a heterogeneous AuNP sandwich immunoassay, which uses immunocolloidal AuNPs [135]. (**b**) Changes in the spectral peak across different HBsAg concentrations (ranging from 1 pg/mL to 1 μg/mL HBsAg) following interaction with the chip. The inset image displays the immunological interaction occurring at the LSPR sensor chip’s active site, utilizing 15, 30, and 50 nm immunocolloidal AuNPs to amplify the signal. HBsAg levels ranging from 1 ng/mL to 100 fg/mL were evaluated using the LSPR chip. Each test was conducted six times, presenting the outcomes as an average ± standard deviation. The coefficient of variation (% CV) was maintained below 10% [138]. (**c**) LSPR sensor (**ci**–**civ**) featuring interconnected nano-rings and a fully etched liquid area for cancerous fluid detection [132].

LSPR sensors represent a specific subset of plasmonic sensing technologies, offering high sensitivity in the detection of various analytes at the nanoscale. These sensors utilize the resonance of SPs localized on metallic nanoparticles (NPs) when they are excited through light. The LSPR phenomenon is extremely sensitive to variations in the dielectric properties close to the NPs surface, rendering it an efficient method for identifying chemical and biological materials [127,128,129]. In LSPR, phenomenon-coherent oscillations of electrons occur at the boundary between metallic NPs and the surrounding dielectric medium. Unlike SPR, which occurs in continuous metal films, LSPR is characterized by its confinement to nanostructures. The resonance frequency of these plasmons is highly responsive to the dimensions, configuration, and composition of the NPs, as well as to the RI of the surrounding medium. The binding of analytes to the NPs surface causes a shift in the resonance frequency, which can be detected optically [60]. Au and AgNPs are commonly used due to their strong plasmonic responses [130]. The choice of the material, size, and shape of the NPs can be tailored to optimize the sensor for specific applications. NPs can be immobilized on various substrates or dispersed in a solution. The substrate material and design play a prominent role in the sensor’s performance. These LSPR sensors have shown promising potential for cancer detection, leveraging their high sensitivity to molecular interactions for the identification of cancer biomarkers. These biomarkers can be proteins, DNA mutations, or other molecules associated with cancerous cells. LSPR sensors detect these biomarkers by monitoring changes in the RI near the sensor surface, which affects the resonance condition of SPs on metallic NPs. To enhance selectivity for specific cancer biomarkers, the surface of the NPs is often modified with recognition elements, such as aptamers, antibodies (Abs), or peptides, that have a high affinity for the target biomarkers [131,132]. Cancer detection often requires the simultaneous detection of multiple biomarkers, and LSPR sensors are designed for multiplexed detection. By functionalizing different areas of the sensor surface with different recognition elements, they thus act as a promising tool for cancer detection [128,133,134]. *Na* et al. [135] in Figure 7a illustrates the approach to enhancing the detection of miRNAs using 3D nanostructures on Au strips, which can be used to attract cancer biomarkers in the signal-amplification process for miRNA detection. Three-dimensional plasmonic Au nanostructures are designed; they involve roll-to-roll nanoimprint lithography (R2R NIL) and subsequent Au deposition. The Au strips were produced through thermal evaporation at a 35° angle onto polyurethane acrylate (PUA) nanograting, which was fabricated using R2R NIL with a polydimethylsiloxane mold, achieving a 200 nm spacing and 100 nm-high nanograting pattern. In the evaluations, these Au strips demonstrated a capacity for high-throughput analysis, and due to their flexible and transparent 3D nanostructure, they offered several advantages for straightforward incorporation into various point-of-care (POC) and lab-on-a-chip diagnostic tools. Moreover, the Au nanostructures achieved via Au deposition on both the top and sidewalls of the PUA nano-gratings at a controlled oblique angle facilitated the creation of SPs with a longer oscillation length. This configuration is recognized for its superior sensitivity to changes in the RI, surpassing that of rectangular rod-shaped Au structures deposited solely on the top. Therefore, through this signal amplification approach implemented on the Au nanostructure sensitive detection of the target in a buffer solution by generating reproducible signals with a distinct LSPR shift is achieved. *Takemura* et al. [126] presented an LSPR-based sensing setup with the potential to detect various viruses via biomarkers based on changes in the local RI at the nanoscale as an optical phenomenon. The approach used to assemble LSPR sensors mirrors that of SPR sensors. It is crucial to alter the material for efficient target capture via nanomaterials while preserving the NPs’ structural integrity. The displacement in the plasmon resonance peak due to the LSPR from plasmonic particles depends upon virus attachment, which serves as a highly sensitive indicator [136,137].

*Kim* et al. [138] present an optical absorbance peak-shift methodology in Figure 7b in which they crafted a design where a virus is encapsulated between two differently sized AuNPs on a substrate enriched with AuNPs. In their configuration, two closely situated AuNPs exhibit repulsion in a plasmonic resonance condition when a virus is present, leading to a more pronounced peak shift than in configurations without the sandwich structure. The signal amplification resulting from this sandwich architecture was validated, achieving a sensitivity enhancement by a factor of 100 compared to cases where the virus is only captured on an AuNPs-rich substrate. Additionally, the sensitivity within the sandwich framework varies with the size of the AuNPs at the site of the secondary antibody. Smaller particles yield a stronger signal, even at lower concentrations of the sample. Their investigation highlights the critical role of particle size in enhancing LSPR-based system development for specific applications. The metallic NPs can be synthesized through various physical and chemical methods, including electrochemical processes, vapor deposition techniques, the decomposition of organometallic compounds, seed-mediated growth, photochemical reduction, and reducing metal salts in the presence of stabilizers. These stabilizers determine the shape and size of the NPs. The unique shapes of these nanostructures, such as nanocages, nano-prisms, and aggregates, display diverse colors or spectra due to their complex, non-degenerate properties. Thus, through this detection methodologies, various cancer biomarkers can be identified and detected.

*Abdi* et al. [132] explore an LSPR-based sensor presented in Figure 7c comprising two interconnected AgNPs rings on a $SiO_{2}/Si$ substrate. The resonance peak arises from the concentrated electric field within the elliptical region between the nano-rings. By imagining a carved-out area filled with a solution under this elliptical region, they enhance the interaction between the solution containing cancer cells and the electric field that has infiltrated this space in the $SiO_{2}$ layer. As a biosensor, their designed model demonstrates efficacy in distinguishing various cancerous cells from healthy ones, exploiting the higher RI of cancer-affected cells.

**Figure 8:**
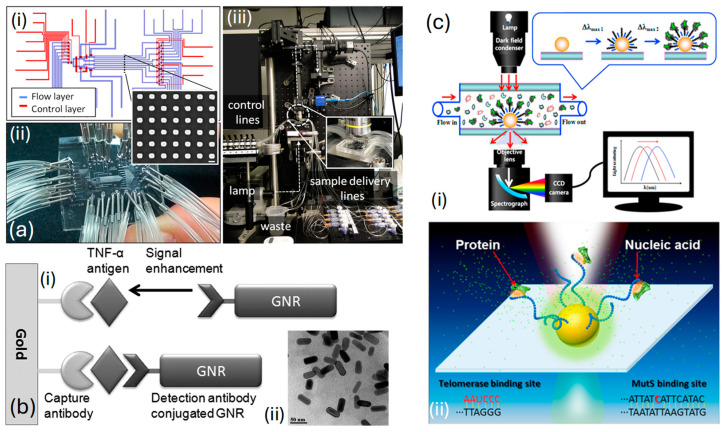
(**a**) (**ai**) Diagrammatic illustration of the flow and control layers that make up the microfluidic chip and the construction of the assembled chip [139]. (**aii**) The inset shows a conventional SEM image of the plasmonic Au sensor [139]. (**aiii**) Developed optical setup [139]. (**b**) (**bi**) Ultrasensitive immunoassay utilizing GNRs as amplification labels [140]. (**bii**) TEM image of a GNR displaying the LSPR peak at 645 nm [140]. (**c**) (**ci**) The setup involving a dark-field microscope combined with a Rayleigh light-scattering spectroscope [141]. (**cii**) Interactions between nucleic acids and proteins inside the sensor [141].

*Acimovic* et al. [139], in Figure 8a, display an SEM image illustrating a segment of a typical nanorod array. To maintain the independence of each NPs array, accurate delivery, and the separation of samples across arrays, are achieved through a microfluidic interface crafted from polydimethylsiloxane (PDMS) polymer. This interface is regulated via micromechanical valves (MMV), which allow for the toggling of microfluidic network functions among different operational modes necessary for the sensor’s preparation and sample analysis stages. The PDMS chip is fabricated and positioned on top of the plasmonic glass substrate, ensuring that the distributed nanoparticle arrays are located within specified areas across eight separate channels, as shown in Figure 8(ai). After securing a strong adhesion between the PDMS and the glass substrate, the device is set for attachment and linkage to the controlled liquid delivery module, illustrated in Figure 8(aii). The optical system for monitoring the chip includes a custom-built microscope designed for bright-field transmission, featuring scanning detection capabilities. It is paired with a visible-near-infrared (VIS−NIR) light source and a spectrometer, as shown in Figure 8(aiii). Thus, through their specially crafted system, they can swiftly identify cancer biomarkers like human alpha-fetoprotein and prostate-specific antigen.

*Law* et al. [140] presented a biosensor enhanced via NPs, merging NPs and immunoassay sensing techniques within a phase interrogation SPR system capable of detecting antigens at extremely low concentrations, down to the femtomolar range. Their research highlights the significant enhancement in sensitivity achieved by extending the plasmonic field from the Au film to Au nanorods (GNRs). Utilizing antibody-functionalized sensing films in combination with antibody-conjugated GNRs. Then, a highly effective plasmonic coupling system capable of serving as a powerful ultrasensitive sandwich immunoassay for detecting cancer-related diseases is established. Figure 8(bi) represents an illustration of an ultrasensitive immunoassay employing GNRs as amplification labels. The TEM image presented in Figure 8(bii) depicts the GNRs utilized in their research. The average dimensions of the GNRs were estimated to be 46.5 ± 2.9 nm in length and 21.1 ± 1.8 nm in width, resulting in an aspect ratio of 2.20. This characteristic is further elucidated by the longitudinal peak observed in the absorption spectrum.

*Ma* et al. [141] introduced a unique nano-plasmonic sensing platform that leverages LSPR for the label-free and real-time identification of highly reliable cancer markers, including mutant genes and telomerase, within clinical samples, as depicted in Figure 8(ci). Their sensor exhibits specific detection of mutant DNA and can detect telomerase from as few as 10 HeLa cells, as represented in Figure 8(cii). This flexible method offers the potential to identify a wide range of pathological markers with outstanding sensitivity and specificity and for observing crucial biomolecular interactions, including those between nucleic acids and proteins as diseases progress in real time. Additionally, their system shows significant promise for advancement, facilitating on-chip analysis and the concurrent detection of numerous targets and interactions.

### 3.3. Classification of Colorimetric Sensors

**Figure 9:**
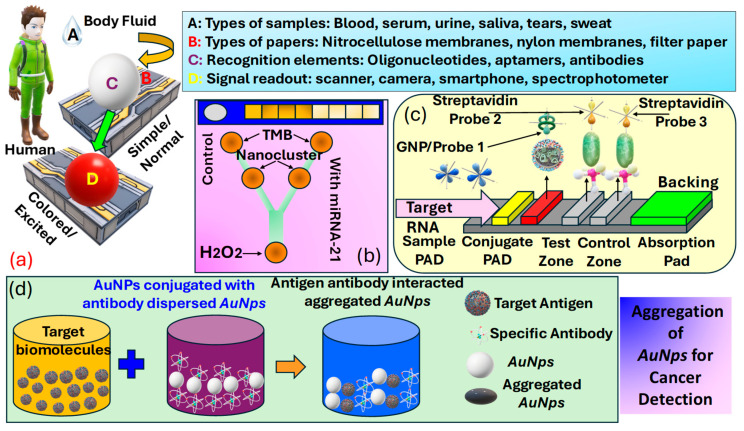
(**a**) Classification of various elements used in colorimetric sensors, such as samples, biomarkers, papers, recognition elements, and signal-readout devices [150]. (**b**) Categorization of PADS testing kits for analyzing urine samples and a microfluidic detection system that can identify microRNA-21 concentrations as low as 1000 pM, employing the peroxidase-mimicking activity of DNA-templated Ag/Pt nanoclusters [151] (**c**) LFA setup for microRNA-215 detection, along with optical measurements [152]. (**d**) Methodology for the preparation of AuNps for cancer cell identification [153].

Colorimetric sensors for cancer detection utilize the principle of color change in response to specific biomarkers associated with cancer, offering a simple, cost-effective, and easily interpretable method for early diagnosis and monitoring. These sensors often involve the use of NPs, such as Au or Ag, which exhibit a change in their plasmonic properties when bound to cancer-related molecules, leading to a visible color shift. The interaction between the sensor and target molecules can be engineered to be highly specific, targeting a wide range of cancer biomarkers, including proteins [142], DNA mutations [143], and microRNAs [144]. The simplicity of observing color changes without the need for sophisticated equipment makes colorimetric sensors particularly appealing for POCT and resource-limited settings. Progress in nanotechnology and biochemistry has markedly improved the sensitivity and specificity of these sensors, positioning them as a promising approach for non-invasive cancer detection [145], assisting in the early detection of tumors [146], and potentially improving patient outcomes through enabling timely treatment. Figure 9a presents details about various elements used in a colorimetric sensor for detection purposes, which include body-fluid samples [6], a variety of papers [147], recognition elements [148], and signal read-out devices [149].

Biosensors are analytical tools that feature a detection element, often referred to as a bioreceptor, e.g., enzymes, antigens, Abs, cells, or nucleic acids, crafted to detect specific targets, and a transducer such as optical, electrochemical, thermal, or mass-based systems tasked with transforming the recognition occurrence into a quantifiable signal. Offering high specificity, biosensors excel at detecting single biomarkers or sets of biomarkers, even at low concentrations. Consequently, researchers have emphasized the advancement of biosensor-based methodologies for disease detection, such as cancer, particularly when they can be engineered into a POCT format, enabling testing outside the conventional laboratory setting. While POCT devices are not designed to completely substitute clinical tests, they have attracted significant interest lately, especially for diagnostic uses, because they provide straightforward and quick initial screenings [150]. *Carneiro* et al. [154], in Figure 9b, present PADS kits and microfluidic detection systems capable of producing rapid results following a noninvasive detection methodology. These devices can be used effectively for cancer detection due to easy detection methodology. Thus, numerous diagnostic assays for cancer biomarkers are available on the market, enabling rapid and non-invasive detection. Figure 9c presents the operational principle of the lateral-flow assays (LFA) for analyte detection. This analyte can also be human body fluid for cancerous cell detection. In addition to these other different technical approaches to the development of PADS based sensors, which include dipsticks, spot tests, and µPADS [153]. Biorecognition elements concerning PADs for cancer biomarker detection are summarized in Table 2. Another important procedure for cancer identification includes the domain of metallic NPs. The dimension of these NPs is often less than 100 nm. In NPs, the unrestricted movement of free electrons within their confined space due to size limitations naturally leads to oscillation at a specific frequency. When these NPs are irradiated with a frequency matching the inherent oscillation frequency of electrons, they absorb energy, inducing a plasmonic effect characterized by non-propagating oscillations. This phenomenon is predominantly observed in metallic NPs. Additionally, bimetallic NPs, composites, and metal oxides also exhibit the plasmonic effect suitable for detecting cancer biomarkers. *Akshaya* et al. [155] conducted a study on the diagnosis of breast cancer-cell MCF-7 utilizing aptamer–cell interactions. MCF-7 cells possess nucleole receptors, which are overexpressed in cancer cells, allowing them to capture nucleole aptamers (AS 1411). When all the AS 1411 bind to the nucleole receptors, the addition of complementary single-stranded DNA-coated AuNPs (ssDNA−AuNPs) results in no remaining aptamers available for binding. Consequently, no aggregation of AuNPs occurs, resulting in the absence of a noticeable color change. In contrast, in normal cells, ssDNA−AuNPs bind with excess unbound aptamers, promoting aggregation and producing a blue color. This phenomenon is observed through spectrophotometry to guarantee a precise colorimetric response that differentiates between cancerous and non-cancerous cells, with a detection threshold of 10 cells. Thus, they were able to demonstrate the ability of their device in the effective detection of early-stage cancer. The procedure of preparing AuNps for cancer cell identification is presented in Figure 9d.

Table 3 summarizes the utilization of the NPs for the detection of cancer biomarkers. Among the diverse types of NPs demonstrated, AuNPs are favored due to their numerous advantageous characteristics, rendering them a promising candidate for sensing applications. The key factor for successful colorimetric detection is robust SPR activity, which directly impacts the sensitivity of the colorimetric sensor.

**Figure 10:**
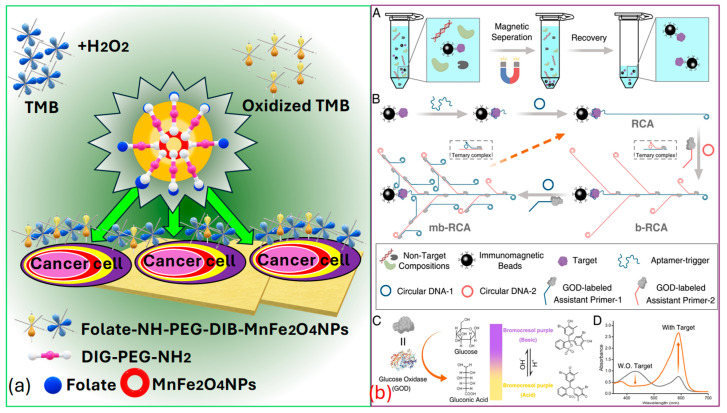
(**a**) Cancer cell identification using MnFe_2_O_4_ [154]. (**b**) Cancerous biomarker identification process by analyzing UV-VIS spectrum. (**bA**) Separation of a target analyte using magnetic beads coated with antibodies (**bB**) Boosting of signal using multi-branched rolling circle amplification (mb-RCA), to increases the amount of glucose oxidase. (**bC**) Illustration showing the reaction caused by glucose oxidase leading to a color change, causing due to pH changes and is detected through color indicator bromocresol purple. (**bD**) UV–visible light absorption results measured both with and without the target protein [155].

*Peng* et al. [154] successfully identified targeted cancer cells using $Mn{Fe}_{2}O_{4}$ as a novel, sturdy, and effective signaling probe. Folic acid (FA), which is widely recognized as a receptor-mediated targeting ligand, exhibits a strong affinity towards FA receptors present on the cell surface of FA-positive tumors. These FA receptors are often overexpressed on the surfaces of certain human tumors, such as ovarian, brain, endometrial, kidney, and breast cancer cells. Thus, by covalently linking FA to NPs, they selectively target various kinds of tumors, as represented in Figure 10a.

In Figure 10b, Miao et al. [155] present the process for the pH colorimetric-based detection of the cancer biomarker protein PDGF−BB that involves several sequential steps. Initially, they target the separation that occurs based on the utilization of immunomagnetic beads. Subsequently, multi-branched rolling circle amplification (mb−RCA) is employed, along with glucose oxidase (GOD) enrichment. The next step involves signal conversion utilizing GOD and pH based colorimetric analysis, facilitated via the pH indicator bromocresol purple (BCP). Finally, the UV−VIS spectrum is recorded with and without the presence of the target protein, providing insight into the detection process. Thus, it is the effective establishment of this pH-dependent colorimetric strategy that holds significant value in advancing the practical utilization of pH in bioanalysis. Moreover, it stands poised to offer insights into the advancement of additional high-performance sensor technologies and is used for cancer biomarker identification. Indeed, recent advancements have demonstrated the efficiency of these biosensors in distinguishing between healthy individuals and patients with various diseases, achieving high classification rates. A study conducted by Moon et al. [165] introduced a bioinspired M13 bacteriophage-based photonic nose capable of differential cell recognition. This sensor presents characteristic color patterns in response to specific biomarkers, enabling the successful identification of different molecular and cellular species. The versatility and tunable selectivity of the M13 bacteriophage make it a promising tool for developing target-oriented sensors with high accuracy and throughput. Additionally, research has shown that M13 bacteriophage-based color sensors can detect cancer cell types by analyzing volatile organic compounds (VOCs) produced via the cells. These sensors demonstrated significant sensitivity and selectivity, with principal component analysis accounting for 99.8% of the variance, indicating excellent discrimination capabilities [166].

### 3.4. Classification of Fluorescence-Based Optical Sensors

**Figure 11:**
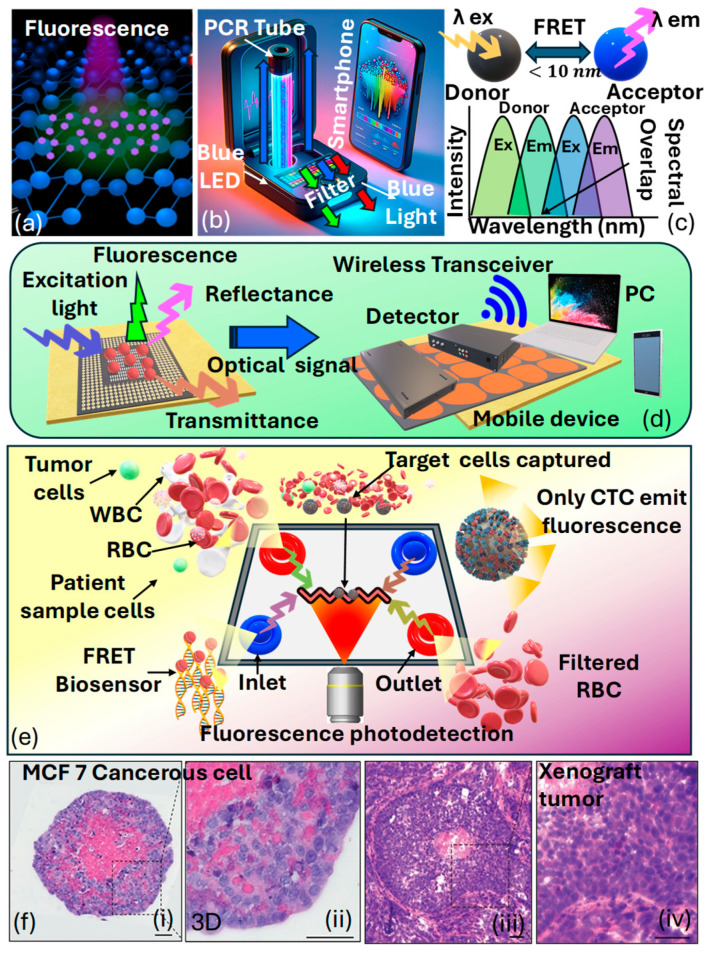
(**a**) Visual appearance of fluorescence NPs [169]. (**b**) Smartphone-based prototype of the fluorescence sensor [170]. (**c**) Condition of the fluorescence resonance-based energy transfer scheme [171]. (**d**) A wireless fluorescence optical biochemical sensor with a mobile optical sensing facility for wireless readout, allowing for direct observation via the naked eye or through remote camera access [172]. (**e**) FRET-based optical sensing platform for cancer cell identification [173]. (**f**) Staining of MCF7-C3 spheroids and xenograft tumors originating from MCF-7 cells using hematoxylin and eosin (i) MCF-7 C3 cancerous spheroid showing compact cell morphology and dense nuclei (ii) 3D culture of MCF-7 cells, exhibiting partially organized structures and intercellular spaces (iii) Tumor section from MCF-7-derived xenograft, demonstrating in vivo tissue organization and vascularization (iv) Higher magnification of xenograft tumor, revealing dense cellular proliferation and nuclear atypia [175].

Fluorescence-based optical sensors are based on the phenomenon of fluorescence, which occurs due to molecules called fluorophores [167]. These molecules capture light energy and then release light at a longer wavelength. In fluorescence-based optical sensors, a specific analyte or target molecule interacts with a fluorescent probe, leading to a change in fluorescence intensity, lifetime, or wavelength. This change is then detected and quantified using optical instrumentation, such as fluorescence spectrometers or microscopes. By measuring the fluorescence signal, the concentration or presence of the target analyte can be determined with high sensitivity and specificity [168]. Fluorescence-based optical sensors can be used effectively to identify cancerous cells in the human body. Specifically, there are three categories of optical wireless sensors utilizing the fluorescence phenomenon, encompassing wearable optical sensors, radio-frequency optical sensors, and smartphone camera-based sensors. Figure 11a presents the artistic view of the visual appearance of fluorescence NPs [169].

*Ponlakhet* et al. [170] presented an efficient approach for the fluorescence detection of $NO_{2}–$ using a portable fluorescence platform based on smartphones. Images captured via the smartphone camera are transmitted to a mobile (Android) application, where the RGB components of the images are analyzed. The obtained fluorescent species (DAP) originates after the reaction between $NO_{2}–$ and CTAB−AuNPs, yielding ${Au}^{3+}$ that acts as a homogeneous catalyst for the oxidation of OPD in the presence of $H_{2}O_{2}$, resulting in the generation of fluorescent DAP. Remarkably, the micelle model of a nonionic surfactant (TX−100) enhances the fluorescence emission of DAP. The RGB intensities of DAP are analyzed to correlate with the concentration of $NO_{2}–$. Moreover, key factors influencing sensitivity are thoroughly examined and refined. The usability of this fluorescence sensing system is showcased through the analysis of pretreated fruit juice and malt beverage samples using a streamlined sample preparation technique. The precision of their assay matches that of the conventional method for $NO_{2}$—analysis. By integrating fluorescence signal analysis with a fluorescence amplification approach on a compact, smartphone-based fluorescence device that includes mobile applications, the complexity of analysis is substantially minimized. This approach is cost-effective, easy to use, and portable, enables swift on-the-spot detection, and offers outstanding sensitivity and specificity for $NO_{2}$—detection. Figure 11b presents the prototype of their proposed fluorescence-based sensor model. Figure 11c presents the condition of the fluorescence resonance-based energy transfer (FRET) scheme used in the fluorescence-based sensor models [171]. *Mohammadi* et al. [172] emphasized wearable sensors that employ fluorescence, combined with smartphones, potentially serving functions such as data storage, image processing, illumination, and radio communication. The merging of wireless sensors with smartphones is particularly viewed as an encouraging advancement in the evolution of fluorescence-based sensors. Figure 11d presents a prototype of the fluorescence-based optical biosensing device that can further be used for cancer cell detection.

*Hernandez* et al. [173] presented FRET-based optical sensors that offer the capability of detecting minute changes ranging from angstroms to nanometers. Nevertheless, traditional fluorescent molecules like organic dyes pose limitations due to their potential toxicity and susceptibility to photobleaching. The integration of nanomaterials into the production of fluorescent devices has surpassed prior challenges, resulting in the creation of affordable, portable sensors that display improved fluorescence signals. This is achieved by utilizing nanostructures that magnify fluorescence signals. Consequently, the use of FRET assays and fluorescence lifetime flow cytometry has made it possible to detect phosphorylated EGFR in tumor cells [174]. These findings represent progress in the advancement of devices capable of capturing subcellular phenomena pertinent to cancer research. Figure 11e represents a schematic depiction of a microfluidics device designed for biosensing utilizing the FRET technique for cancer detection.

*Anand* et al. [175] noticed that the limited diffusion of nutrients and waste products beyond 150–200 μm, crossing cellular barriers, leads to hypoxic conditions, ultimately resulting in the fragmentation of the nucleus and necrosis within the spheroid. This observation was clarified using H&E staining on a spheroid formed via seeding 10,000 MCF7−C3 cells, which was then cultured for 5 days, as shown in Figure 11f. The lack of hematoxylin staining in the nuclei at the spheroid’s core highlighted necrotic cells in that central area. In contrast, hematoxylin staining in the spheroid’s outer region (∼200 μm) indicated that the nuclei were preserved in these cells. Additionally, the morphological features and the detection of a necrotic core in the histological sections of the xenograft tumors highlight that the spheroids faithfully mimic the histological attributes of tumors in a living organism. Consequently, various strategies can be employed to detect different cancer biomarkers using fluorescence-based optical biosensors.

Table 4 provides details on biomarker detection in cancerous cells using FRET technology. It highlights several biomarkers, such as mRNAs and specific cancer-related proteins, along with their respective detection methods across different types of cancers.

### 3.5. Classification of Photonics and Waveguide-Based Optical Sensors

Photonics-based optical sensors are utilized in cancer detection because of their high sensitivity and specificity, along with their capability to identify minor changes in biological samples that are indicative of cancerous growth. These sensors operate on the principle of exploiting the interaction between light and the target analyte to generate measurable signals. In photonics-based optical sensors, light is used to probe the sample, and changes in the optical properties of the sample, such as absorption, fluorescence, or RI, are monitored to detect the presence of cancer biomarkers [186]. On the other hand, waveguide-based optical sensors use waveguides, such as OF or planar waveguides, to confine and guide light through the sample. Changes in the optical properties of the sample within the waveguide, such as RI or absorption, can be detected by monitoring changes in the guided light signal. These sensors are extremely sensitive to variations in the sample, allowing them to detect cancer biomarkers at very low concentrations [187].

**Figure 12:**
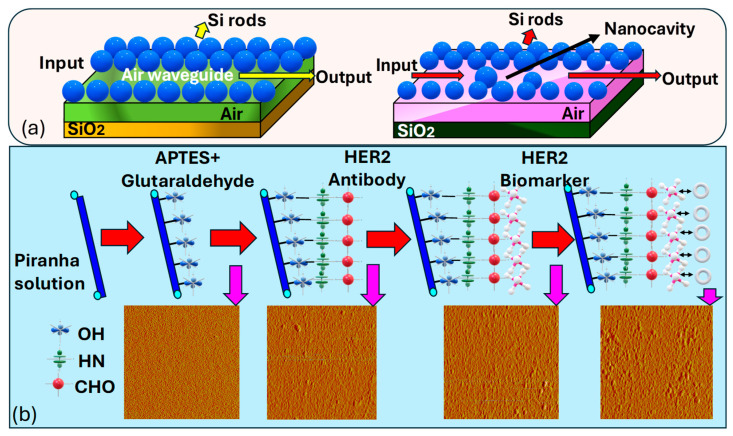
(**a**) Three-dimensional (3D) visualization depicting the dielectric profile of a linear air waveguide formed via the removal of a row of dielectric holes. Parameters include a lattice constant of a = 0.8 μm, air-hole radius of r = 0.3a, Si rod length of 1.5 μm, and SiO_2_ layer thickness of 3 μm and the depiction illustrating the dielectric profile of a nanocavity-coupled waveguide structure [188]. (**b**) Illustrates the conjugation and the morphology process of the fiber surface using AFM. Ultimately, following the detection of the HER2 biomarker, the fiber surface showed the highest level of roughness, marked by the agglomeration of bio-particles [189].

*Jindal* et al. [188] introduce a nanocavity-coupled photonic crystal waveguide (PCW) based biosensor serving as an on-chip platform for the label-free identification of cancerous cells, as represented in Figure 12a. Their proposed biosensing platform demonstrates remarkable sensitivity and a high Q. In their platform, they have used label-free optical biosensing, which provides a straightforward sensing approach by eliminating the need for fluorescent labeling of analyte molecules. The incorporation of a silicon (Si) nanocavity within the PCW is used, which offers advantages over 2D-PC micro-cavities, providing a much smaller sensor area while maintaining sensitivity. Monitoring the resonant wavelength (RW) shift or tracking the intensity variation at a specific wavelength sensing mechanisms is available in the proposed sensor. The integration of nanocavity in the PCW enhances the capability of the chip for cancer cell detection. Optimizing the structure of the inserted nanocavity sharpens the resonant peak of the output spectra, enabling customizable and highly sensitive detection of cancer cells. The binding of molecules with different cancer cells induces shifts in the observed peaks in the transmission spectra to distinct wavelengths.

Sun et al. [189] presented a covalent immobilization procedure, as illustrated in Figure 12b, for preparing the sample solution to cancer cell identification. Firstly, they treated a freshly prepared piranha solution $\left(H_{2}{SO}_{4}/H_{2}O_{2}=70:30,v/v\right)$ for 30 min, followed by rinsing with de-ionized (DI) water and nitrogen drying. Then, reacting the fiber surface with 5% 3−aminopropyl−triethoxysilane (APTES) in ethyl alcohol for 15 min, thorough rinsing with DI water, and subsequent drying are performed. Then, a crosslink reaction using a 2.5% glutaraldehyde solution for 30 min, followed by thorough rinsing with DI water and drying, is performed. Then, immobilization of the HER2 antibody with a concentration of 10μg/mL for 1 h is performed, followed by thorough rinsing with DI water.

The final step involves immersing in a 1% bovine serum albumin (BSA) solution in phosphate-buffered saline (PBS) for 5 min to block unreacted sites and reduce nonspecific adsorption. Employing piranha solution is essential in creating reactive hydroxyl groups on the surface of the fiber. Following this, applying fresh 3−APTES in ethyl alcohol aids in forming free amine groups NH2− on the fiber surface, thus efficiently immobilizing it. This salinized fiber was then primed for further reaction with aldehyde groups (−CHO) present in glutaraldehyde, leading to the formation of imines. Following this, the fiber underwent a 2 min rinse with DI water to eliminate excess adsorbed components, followed by drying with nitrogen. Subsequently, the fiber was immersed in a solution containing HER2 Abs for 60 min and rinsed with DI water thereafter. This process ensured the elimination of non-reacted sites, thereby minimizing nonspecific adsorption when immersed in the BSA solution. As a result, the functionalized biosensor probe was prepared for the detection of HER2 biomarkers responsible for breast cancer.

**Figure 13:**
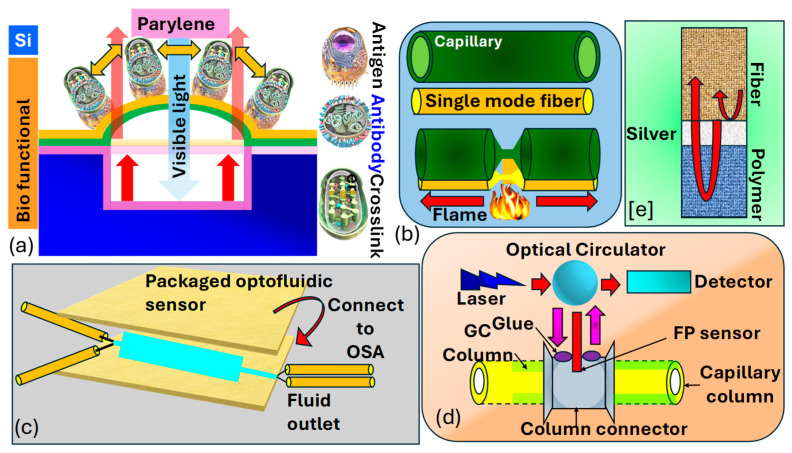
(**a**) Illustration depicting a MEMS optical interferometric surface-stress immunosensor in cross-section [190]. (**b**,**c**) Configuration of optofluidic biosensor for biomolecular detection [191]. (**d**) FP sensor model combined with a GC column, light source, and detector [192,193]. (**e**) Showcasing the FP sensor created through the sequential application of Ag and polymer coatings to the fiber end [192,193].

Madea et al. [190] illustrate the structural layout and operational principle of the optical interferometric surface-stress sensor, as shown in Figure 13a. The proposed sensor consisted of a thin layer of parylene−C with a bio-functional coating, mimicking a flexible membrane situated above a cavity formed on a Si substrate. Hence, the Fabry–Perot interferometer (FPI)  comprised a flexible membrane, an air gap, and a Si substrate. The interaction between antigens and Abs led to the deformation of the membrane due to repulsive forces arising from charged antigen binding. As the surface stress caused the flexible membrane to bend, the distance between the Si substrate and the distorted membrane expanded, leading to a spectral change towards longer wavelengths. Consequently, spectral shifts allowed for the assessment of nano-mechanical deflection of the flexible membrane. To heighten detection sensitivity in the surface-stress sensor, one can utilize a soft material characterized by a low Young’s modulus and a thin film. This approach leverages the inverse relationship between the deflection magnitude of the sensor and the material’s Young’s modulus, as well as the thickness of the film. In their investigation, parylene−C is selected as the support layer for the freestanding membrane due to its significantly lower Young’s modulus, which is two orders of magnitude less than that of Si, and its capability to form uniform nanosheets of submicron thickness through CVD at 25 °C.

*Liang* et al. [191] demonstrated the fabrication process of the optofluidic sensor presented in Figure 13b,c. They took a bare 125−µm single-mode OF precisely aligned with a Si capillary, ensuring lateral contact and alignment in the same direction. The Si glass capillary possesses an outer diameter of 660 µm and a thickness of 65 µm. By employing a flame as the heat source, both the fiber and the capillary underwent substantial tapering, shrinking their dimensions by tens of times. This procedure entailed heating the Si glass to its softening point while gradually stretching it with two fiber holders. The resultant tapered configuration consists of a microfiber and an extremely thin capillary, enabling efficient light penetration and interaction with the fluid enclosed within the capillary. This configuration can be effectively utilized as an optofluidic sensor for biomolecular detection. *Khan* et al. [193] present an FP sensor utilizing a metal-coated FP cavity to detect VOCs such as hexanol, methanol, and acetone, as shown in Figure 13d,e. The interferometer is constructed by sequentially depositing Ag and a gas-sensing film at the end of an OF, forming the FPI cavity through Ag–polymer and polymer–air interfaces. Norland optical adhesive (NOA−81) and polyethylene glycol (PEG−400) were chosen as the sensing film due to their favorable optical and chemical characteristics. Using PEG−400 as the sensing film, a sensitivity of 3.5 pm/ppm with a detection limit of 1 ppm was achieved. This design was expanded to integrate with gas chromatography (GC) for column detection of decane, toluene, methanol, and dimethyl-methyl-phosphonate (DMMP). The sensitivity of the sensor is improved to sub-nanogram levels, and a detection limit as low as 50 pg was attained for DMMP. The reported sensitivities of their designed sensors appear to be 4.75 mV/ng and 77 mV/ng for decane and DMMP, respectively. Thus, the methodology suggests that, through photonics and waveguide-based sensors, cancer biomarkers can also be detected efficiently.

### 3.6. Classification of Fiber-Optics Sensors

**Figure 14:**
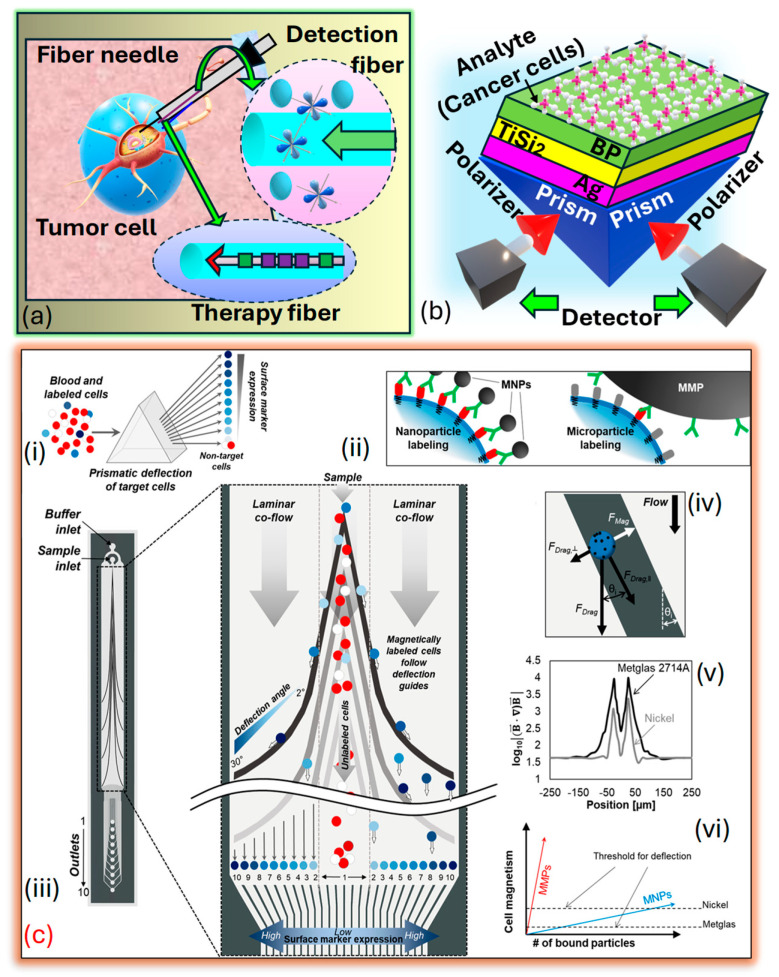
(**a**) The device is a minimally invasive tumor theragnostic needle featuring functional optical fibers designed for tumor navigation. The system integrates an in-situ fiber tumor detector that emits an excitation laser, detects tumor-specific markers, and captures fluorescence signals. Cancer marker-sensitive fluorescent probes situated on the fiber surface interact with hypoxia markers, enabling the fiber to locate tumors for precise identification. This fiber also carries the excitation laser and collects tumor-related fluorescence, enabling precise detection and localization of tumors. Additionally, it contains a photothermal therapeutic fiber with a core-sealed rare-earth-based photosensitizer. The rare-earth elements convert laser energy to heat for targeted, efficient, and safe hyperthermia treatment. A Bragg grating monitors local temperature during therapy, ensuring controlled and accurate photothermal treatment [195]. (**b**) PRISM-based SPR sensor for cancer cell identification [199]. (**c**) Prism chip design. (**ci**) Prismatic deflection segregates a continuous sample flow into distinct subgroups according to the expression of surface markers. (**cii**) Magnetic nanoparticles (NPs) provide a more precise reflection of cell surface protein expression compared to larger magnetic microparticles. (**ciii**) The diagram illustrates the cobalt-based ribbon-prismatic deflection chip, comprising deflection guides composed of individual segments with angles ranging from 2 to 30 degrees. (**civ**) During the analysis of forces acting on a cell in the horizontal plane (excluding friction), magnetically labeled cells follow the deflection guides until the magnetic attraction towards the guide is counterbalanced with the perpendicular component of the drag force against the guide. (**cv**) The assessment underscores the enhancement of magnetic field strength via nickel and cobalt-based deflection guides. (**cvi**) When comparing factors influencing cell deflection, cobalt-based deflection demonstrates a requirement for lower particle concentration to achieve effective deflection compared to similar nickel-based guides [198].

It may sound similar, but fiber-optic sensors differ in design, operation, and applications as compared to *PCF* *SPR* sensors. Fiber-optic sensors broadly measure parameters like temperature, pressure, and strain using light transmission and techniques such as intensity modulation and interferometry. They are versatile, with applications in telecommunications, healthcare, and environmental monitoring. In contrast, *PCF* *SPR* sensors are a specialized subset, leveraging *PCF* and *SPR* for ultrasensitive *RI* detection. Tailored to precision applications like biomolecular interactions and chemical analysis, *PCF* *SPR* sensors excel in biosensing, offering high sensitivity compared to general-purpose fiber-optic sensors. Fiber-optic sensors for cancer detection offer promising avenues for early diagnosis and monitoring of the disease. These sensors utilize the unique properties of light transmission through OF to detect biomarkers associated with cancerous growth or changes in tissues. These fiber-optic sensors offer advantages such as high sensitivity, real-time monitoring capabilities, and minimally invasive detection, making them appropriate for several cancer detection applications. They can be integrated into medical devices for the in vivo or ex vivo detection of cancer biomarkers in bodily fluids, tissues, or cells. Additionally, their compatibility with imaging techniques such as endoscopy or microscopy enables localized and targeted detection of cancerous lesions. Research in fiber-optic sensors for cancer detection continues to advance, focusing on enhancing sensitivity, specificity, and multiplexing capabilities to enable early and accurate diagnosis of cancer, resulting in improved patient outcomes and personalized treatment. *Xu* et al. [194] prepared a fiber-optic interstitial needle presented in Figure 14a designed to deliver hypoxia-sensitive fluorescent probes and encapsulated rare-earth dopants for in vivo tumor treatment, employing a combination of endoscopic cancer sensing and photothermal therapy (PTT). These specialized OF, with compact diameters measuring several hundred microns, can be arranged and inserted side by side into a standard syringe needle, allowing for precise interstitial navigation. The detection fiber, armed with fluorescent probes sensitive to tumor markers, rapidly surveys the area for hypoxia markers within the tumor. Additionally, flexible rare-earth-doped fibers serve as containers for photothermal sensitizers, enabling direct tumor ablation. *Loyez* et al. [195] depict the layered structure arranged in the *Kretschmann* configuration on $Ti{Si}_{2}$. A monochromatic light source of wavelength 633 nm is directed onto the BK7 prism, refracted through the layers, and emerges in the opposite direction, adhering to the principles of TIR. The prism is coated with a thin layer of Ag that has a diameter $D_{1}=45\;\mathrm{nm}$, followed by a two-dimensional (2D) $Ti{Si}_{2}$ nanolayer with a diameter $D_{2}=P*2\;\mathrm{nm}$, where P represents the number of $Ti{Si}_{2}$ films. Subsequently, a BP thin film with a diameter $D_{3}=B*0.5\;\mathrm{nm}$, where B represents the number of BP films covering the $Ti{Si}_{2}\;$layer, serving as an interface between the $Ti{Si}_{2}$ film and the sensing medium. The BP layer enhances the performance of the biomolecular recognition element, which is helpful in cancer cell identification. The BK7 prism functions as a coupling prism in this setup presented in Figure 14b. Several BK 7 prism-based models are presented in literature suitable for cancer detection and early prediction [196,197]. *Aldridge* et al. [198] introduced a microfluidic device engineered to separate magnetically labeled cells and sort them into distinct subcategories according to their surface protein expression. This separation is accomplished using ferromagnetic guides with variable angles, leveraging prismatic deflection technology. This technique partitions a steady stream of cells into individual segments, like how a prism divides light into its constituent wavelengths, as presented in Figure 14c.

*Kaur* et al. [200] proposed a fiber-optic sensor to diagnose different cancers, including blood, cervical, adrenal gland, and breast cancer, at a wavelength of 1550 nm. The sensor model possesses a meticulously designed structure consisting of a fiber core, a clad metal layer, and 2D material, i.e., $MoS_{2}$ and graphene. The sensor offers three optimized probe configurations to enhance sensitivity and overall performance. The FOM indicates $SiO_{2}-PF-Ag-MoS_{2}$ as the most effective probe structure, exhibiting superior sensitivity to RI changes. The sensor’s low LOD values, particularly with $SiO_{2}-PF-Ag-MoS_{2}$, underscore its ability to detect subtle RI variations associated with different cancer cell types, making it a suitable tool for early cancer detection. *Ribaut* et al. [201] introduced an innovative OF immunosensor tailored to detecting cytokeratin 17 (CK17), a pivotal biomarker in lung cancer diagnosis. The sensor’s design enables evaluation in non-liquid environments. Initial experiments involved detecting CK17 within a gel matrix to simulate tissue samples. Au-coated immunosensors, housed a specially engineered packaging for enhanced rigidity, successfully penetrated soft materials, unveiling a stable SPR signal within such substrates for the first time. Further tests targeting CK17 entrapped in a porous polyacrylamide gel matrix underscored the sensor’s selective and specific response to the target protein. Validation through a preliminary examination of human lung biopsy samples confirmed the ex-vivo detection of CK17, marking a significant step towards addressing the clinical challenge of detecting biomarkers in tissues for minimally invasive in vivo medical diagnosis.

The influence of fiber shape on the optical sensor performance is also an important factor to consider while using the fiber sensor for cancer diagnosis. In this section, information about various fiber shapes and their impact on the sensor performance is presented.

**Figure 15:**
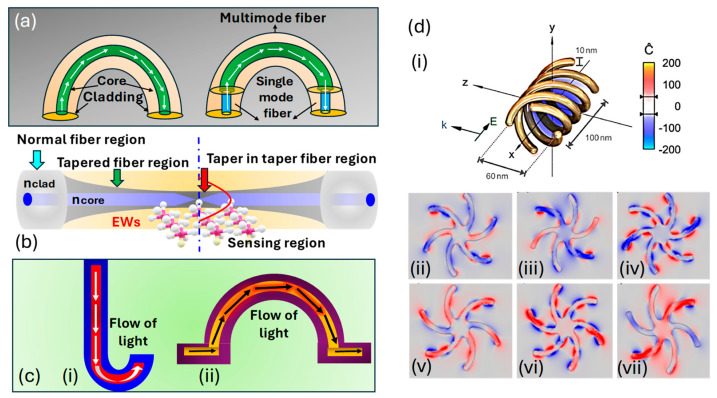
Classification of fiber sensors based on shape. (**a**) U-shaped fiber [203]. (**b**) Tapered fiber [204]. (**c**) (**ci**) J-shaped fiber [207]. (**cii**) Ω-shaped fiber [207]. (**d**) (**di**) Helically shaped fiber. (**dii**) Left-hand circular polarization (LHCP−1). (**diii**) LHCP−2. (**div**) LHCP−3. (**dv**) Right-hand circular polarization (RHCP−1). (**dvi**) RHCP−2. (**dvii**) RHCP−3 [209].

U-shape fiber: The U-shape fiber is designed by bending the optical fiber into a U-shaped curve, as represented in Figure 15a. This shape enhances the light interaction with the analyte by focusing the light directly on the sensing surface. The increased surface area in the U-shaped region allows the interaction of the evanescent field more efficiently with the surrounding medium, which ultimately improves the sensitivity of the biosensor. The impact of the U-shaped curve on the sensor surface can be summarized as follows.

- *Increased interaction area*: the U-shape enables exposure of a larger portion of the light to interact with the surrounding environment, which improves the detection of analytes and binding events [202].
- *Enhanced sensitivity*: the bending of the fiber increases the path length over which light interacts with the sample; this results in enhanced biomarker detection [203].

Tapered fiber: A tapered fiber has a narrow waist along the length of the fiber. This shape enhances the evanescent field by concentrating the light as it travels through the fiber, which results in the development of a strong light interaction with the analyte in the surrounding medium, as represented in Figure 15b. Tapered fibers are commonly used in SPR-based sensors, resonator coupling, and other biosensing applications where sensitivity is ultimately achieved. The impact of the tapered fiber on sensor performance can be summarized as follows.

- *Concentration of light*: the tapered region of the fiber is responsible for focusing the light and increasing the intensity of the evanescent field at the sensor’s surface [204].
- *Enhanced detection*: the improved evanescent field enhances the sensitivity of the sensor to respond against tiny changes in the RI or the binding of biomolecules to the sensor surface [205].

J-shaped and Ω-shaped fiber: The Ω-shaped and J-shaped fibers are characterized by a compact and bent structure that enables integration into small-scale, high-performance optical systems, as represented in Figure 15c. The bending is designed to focus the light on the surface and optimize the interaction with the analyte. These shaped fibers are used in integrated biosensing platforms where space and miniaturization are most important. The impact of the tapered fiber on sensor performance can be summarized as follows.

- *Compactness and integration*: These shape fibers are physically small but quite efficient, making them suitable for use in compact device designs without compromising performance. The small form of these sensors is ideal to be used for portable or wearable diagnostic devices [206].
- *Localized sensing*: The light in these sensors is confined to specific regions of the fiber, allowing for the focused detection of localized binding molecules [207].

Despite having these common properties, Ω-shaped fiber provides more interaction length due to a looped structure, resulting in higher sensitivity, whereas J-shaped fiber, due to its simple structure, is preferred when ease of fabrication or mechanical robustness is required.

Helically shaped fiber: Helically shaped fibers are wound into a spiral configuration and used in fiber-optic sensors mostly for polarization control operations, as represented in Figure 15(di). The coiled structure allows the fiber to interact with the analyte over multiple passes, which increases the interaction area and ultimately increases the sensitivity. These shapes also assist in controlling light polarization, which is helpful for sensing applications, as presented in Figure 15(dii–dvii). The impact of helical fiber on sensor performance can be summarized as follows.

- *Multi-pass interaction*: the helical geometry enables multiple passes of light, increasing the path length and finally the interaction with the analyte [208].
- *Polarization control*: the helical design of the fiber assists in controlling the polarization state of light and improving the sensitivity for molecular interactions [209].
- *Increased surface area*: the helical configuration increases the effective sensing surface area for biomolecule binding, allowing more biomolecules to bind and, therefore, enhancing the sensor’s overall sensitivity and durability [210].

### 3.7. Classification of Raman SERS

**Figure 16:**
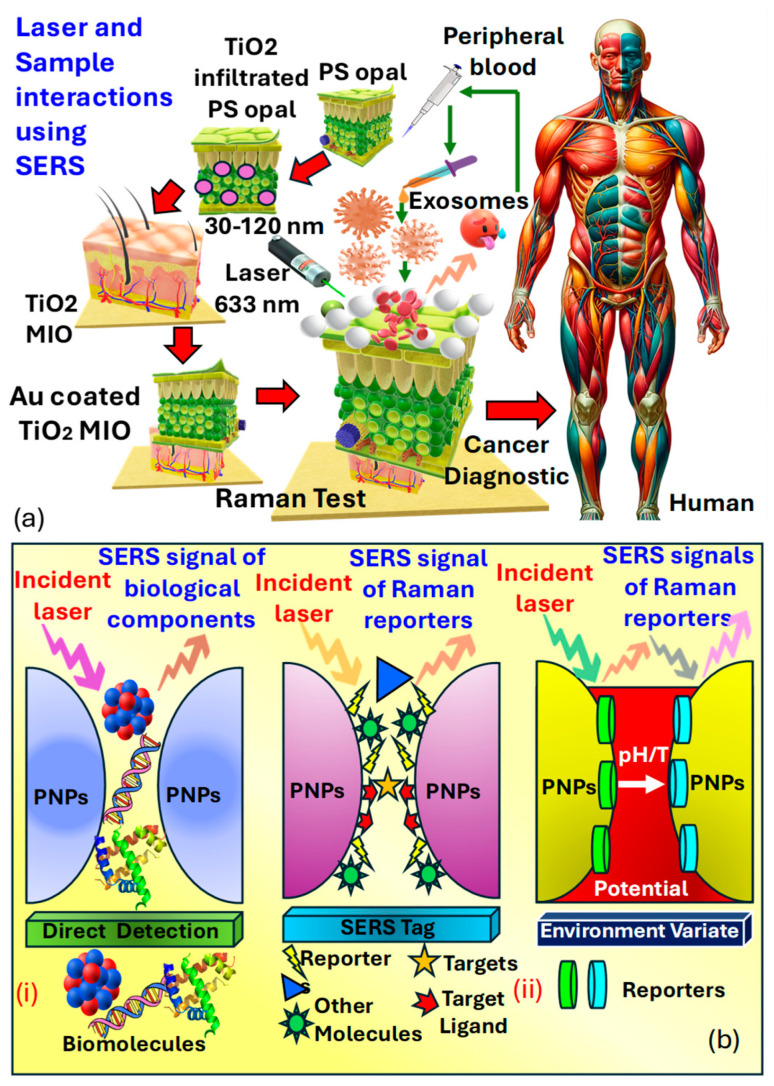
(**a**) A 3D MIO structure, consisting of Au−coated TiO_2_, is developed to enable substantial interaction between the laser and the sample in SERS [211,213]. (**b**) illustrates the design and construction of SERS-based biosensors for both direct and indirect detection approaches. (**bi**) Direct SERS detection entails capturing intrinsic SERS signals of biomolecules from the substrate without requiring labeling molecules [214,215]. (**bii**) Indirect SERS detection utilizes SERS signals from Raman reporters to indirectly indicate the presence of bound targets or changes in environmental properties [214,215]. (PNPs refer to plasmonic nanoparticles.)

Surface-enhanced Raman spectroscopy (SERS) is an analytical technique that involves shining monochromatic light onto a sample and measuring the scattered light, providing information about the sample’s molecular composition, structure, and distribution [72]. Its label-free nature, high sensitivity, and specificity make it valuable for cancer detection, allowing for a real-time analysis of tissues during surgery to ensure complete tumor removal and minimize damage to healthy tissue [211]. Raman spectroscopy can differentiate between normal and cancerous tissues based on their molecular signatures and has been applied to various cancer types for diagnosis, intraoperative margin assessment, and monitoring treatment response. Its ability to detect molecular changes and multiplexing capability offers comprehensive molecular profiling and personalized patient care, with minimal sample preparation requirements [212]. *Harron* et al. [211] investigated the development of a macro-porous SERS probe for cancer detection utilizing EXOs as presented in Figure 16a. Notably, the presence of phosphoproteins within EXOs serves as a key factor in cancer detection. Within the realm of molecular biology NPs composed of proteins, lipids, and nucleic acids, known as EXOs, are prevalent. These particles, enclosed in a lipid bilayer, typically range in size from 35−110 nm. EXOs have emerged as an intriguing source of biomarkers in clinical diagnostics. However, their structural and compositional modifications allow them to mitigate the adverse effects of proteases and other enzymes. Furthermore, the presence of phosphoproteins within EXOs forms the basis for cancer detection.

Sensitive three-dimensional $\left(3D\right)$,$\;Au-TiO_{2}$ microporous inverse opal (MIO) structures were created to enhance interactions between the laser and the sample for precise SERS measurements. A spectral analysis of exosome vesicles in plasma samples from both prostate cancer patients and healthy individuals demonstrated the capability for detecting prostate cancer in biomedical contexts. This was achieved by observing the signal strength of the $1087\;{\mathrm{cm}}^{-1}$ peak, associated with EXOs in plasma. The notable peak at $1087\;{\mathrm{cm}}^{-1}$ in SERS analysis is linked to phosphate bond scattering, which is directly related to protein phosphorylation, thus acting as an efficient marker for cancer diagnosis. It was observed that the SERS peak at $1087\;{\mathrm{cm}}^{-1}$ from EXOs derived from cancer cell lines was twice as intense compared to those from normal cells, rendering it a significant indicator applicable in prostate cancer diagnostics.

*Li* et al. [214] presented an advanced enhancing structure, and substrates have greatly expanded the applications of SERS, particularly in the biomedical and life sciences fields. Two main methodologies exist for constructing SERS-based biosensors, including label-free detection and indirect detection requiring SERS tags. Biosensors utilizing direct detection offer molecular information about biomolecules by directly monitoring their intrinsic vibrations without the need for labeling molecules. In direct SERS sensing, biomolecules are typically concentrated at the boundary of electromagnetic field, which enhances the substrates, as presented in Figure 16(bi), and surface-bound affinity agents are required for molecules unable to remain within this area or present in low concentrations. Nonetheless, direct sensing encounters limitations due to its intrinsic detection mechanism, where signals from specific biomolecules can be relatively low, intricate, and vulnerable to interference from other molecules within the matrix. In contrast, indirect SERS sensing involves labeling specific SERS signals onto enhancing substrates as SERS tags represented in Figure 16(bii). While analyzing various forms of cancers, human body fluids can be effectively used as an analyte in cancer cell detection using the SERS platform. Table 5 presents a summary of information about various human body fluids, the SERS platform, methods, etc., used in cancer detection.

**Figure 17:**
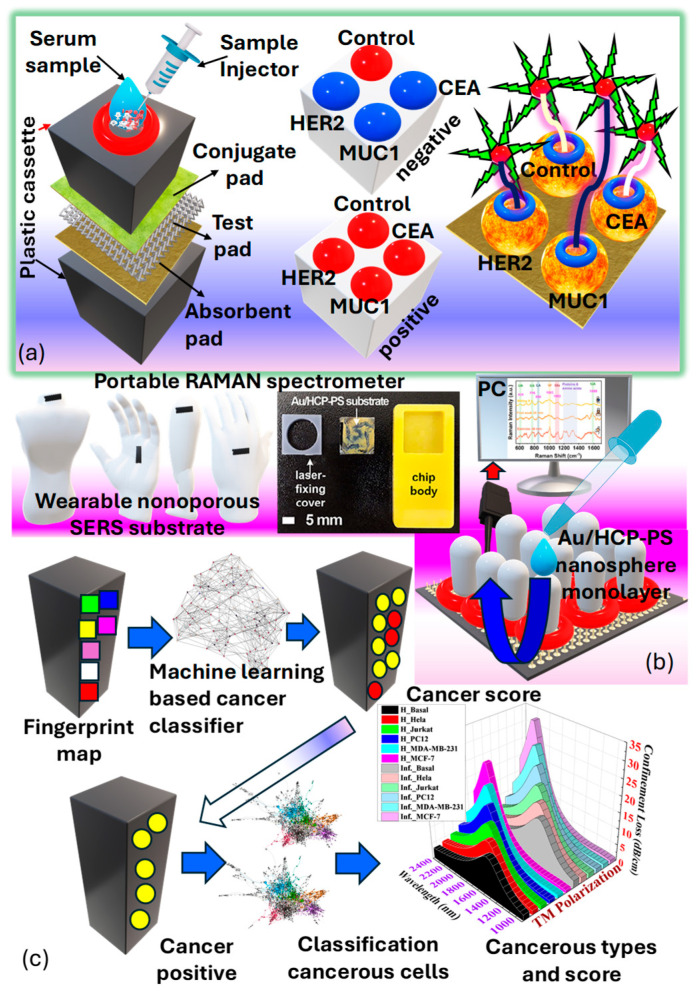
SERS-labeled POCT system designed for identifying EXOs, circulating tumor DNA (ctDNA) and microRNA within body fluids. (**a**) Multiplexed quantitative examination of serological exosome proteins using a combined SERS-VLA biosensor [225]. (**b**) Improved medical health monitoring through a wearable nanoporous SERS substrate that captures and analyzes sweat; this includes the highly sensitive detection and quantification of biomarkers in tears using an onsite, label-free SERS platform for diagnosing breast cancer [226]. (**c**) Simultaneous detection of six early-stage cancer types using machine learning-enhanced SERS analysis [227].

*Hessvik* et al. in [224] suggested that EXOs, which facilitate communication with the tumor microenvironment through the transportation of proteins and nucleic acids, which has emerged as promising biomarkers for early cancer diagnosis, monitoring, and evaluating treatment effectiveness. However, quantitative and phenotypic studies of EXOs are hindered by their low protein content due to their small size. While ELISA is the Au standard for exosome quantification, it struggles with capturing all target EXOs subpopulations and analyzing multiple samples rapidly. Currently, exosome quantification primarily relies on recognizing specific membrane surface proteins, as presented in Figure 17a.

*Tian* et al. [226] presented a flexible, nanoporous, wearable sensor featuring a goldnanostar (AuNS) integrated with an ion track-etched polycarbonate coating as the SERS sensor. This sensor’s nanoporous nature and flexibility enable convenient and effective sweat collection from different body parts. Even under stationary and mild exercise conditions, this epidermal SERS sensor successfully captures spectral information from sweat analytes; the prototype developed by them is presented in Figure 17b. *Shin* et al. [227] presented an AI framework for cancer detection; the initial phase involves assigning diagnostic scores, which are determined by averaging the outcomes of a multiple instance learning-based cancer classifier, as presented in Figure 17c. *Su* et al. [228] demonstrated a quantitative analysis of cancerous EXOs in breast cancer using a SERS-based lateral flow strip (LFS), offering faster detection compared to ELISA due to the operational simplicity of LFS. However, exosome quantification currently provides only quantitative information without distinguishing between different cancer types. Although the mass spectrometry analysis of exosome proteins offers high sensitivity and specificity, it requires sophisticated sample pretreatment and complex instrumentation. To streamline operation steps and reduce analysis times, integrated and malfunctional SERS sensing platforms have been proposed.

*Li* et al. [225,229] proposed multiplexed quantitative profiling of EXOs proteins present in breast cancer patients developed an integrated SERS−VLA platform capable of filtration, injection, and multi-indicator detection within 10 min. Body fluid sweat contains a lot of information regarding human physiological states, as has long been recognized, yet challenges in its collection and analysis have hindered extensive study [230]. However, with advancements in technologies like micronano device fabrication and integrated hardware/software systems, wearable sensors are emerging as a prominent solution for POCT. *Zheng* et al. [231] presented a biosensor that boasts high conformability and biocompatibility to the skin, facilitated by their excellent mechanical flexibility. This flexibility proves invaluable in sweat collection and analysis, leading to the exploration of several compact wearable plasmonic biosensors for label-free detection of sweat-based SERS methods [232,233].

## 4. Summary and Future Perspective

This study concentrates on the advancements in optical biosensing methodologies for the detection of various cancer types. Presently, optical biosensors are prioritizing the enhancement of sensitivity, lowering detection limits, and targeting specific biomarkers for the early detection of cancer. It is worth noting that there is no universal marker for detecting all types of cancers, presenting a challenge in disease detection. Optical biosensors offer convenient access to anatomical areas that are typically hard to reach, such as the thoracic wall. Several successful strategies, including the utilization of NPs, nanocavity structures, 2D materials, and variously shaped fiber cores, have been documented to amplify optical signals and facilitate the detection of low concentrations of diverse biomarkers and cancer cells. Each type of optical biosensor offers distinct advantages and drawbacks. Plasmonic biosensors, including SPR, LSPR, and SERS, boast advantages such as rapid, real-time, and label-free detection. However, LSPR biosensors are prone to interference from nonspecific species, potentially yielding false results in complex environments. Although SPR and LSPR biosensors are commercialized, they suffer from limited specificity, detection limits, and signal-to-noise ratios, requiring large and complex equipment [61]. Colorimetric biosensors are readily available commercially, offering simplicity, rapidity, portability, and cost-effectiveness without the need for sophisticated analytical tools. However, these qualitative sensors often exhibit limited sensitivity and multiplexing capabilities. Conversely, interferometric biosensors, for instance, struggle with challenges such as low coupling efficiency, packaging issues, and precise alignment requirements [150]. Fluorescence biosensors encounter difficulties in pinpointing specific targets due to the inherent fluorescence of tissues or cells, which can generate background signals. Additionally, maintaining a clear solution is necessary to prevent interference among molecules. The complexity and cost escalate due to the necessity of components like filter fluorometers and spectrofluorometers. Furthermore, controlling the binding site of fluorescent molecules presents challenges, potentially leading to false results through interactions with target molecules. Drawbacks such as limited multiplexing, prolonged assay times, photobleaching, and phototoxicity further hinder fluorescence-based approaches [234]. Electrochemiluminescence-based sensors offer high sensitivity, simplicity, miniaturization potential, stability, rapidity, disposability, and controllability. However, they may produce false positives or negative results due to intensity fluctuations. Challenges such as high background noise, environmental interference, low sensitivity, detection limits, and specificity hinder the widespread clinical and practical application of optical biosensors [235]. In waveguide and interferometer sensors, it is important to note that the interferometer sacrifices potential information available from independent TE and TM modes to achieve a lower detection limit for changes across the surface area. Moreover, the realization of a fully integrated difference interferometer poses challenges due to the robustness and integration difficulties associated with the components. The requirement for end-fire coupling to excite the modes and the detection relying on the interference pattern created off-chip necessitates high mechanical stability of the device, potentially limiting its suitability for POC applications [236]. SERS-based sensors excel in detecting analytes in opaque solutions like blood or urine with single molecule-level sensitivity, specificity, and multiplexing capabilities. Despite their advantages, SERS biosensors face challenges such as substrate degradation over time, the need for spectral analysis software, and bulky optical components. Both SERS and fluorescence techniques offer ultrahigh sensitivity, enabling single molecule detection without the need for labeling [237]. The prospects of optical biosensors are vast and promising across numerous fields. In pharmacy and medicine, optical biosensors hold the potential to revolutionize drug discovery, personalized medicine, and the real-time monitoring of therapeutic efficacy [238,239]. Food and beverage inspection and safety stand to benefit from the rapid and sensitive detection capabilities of optical biosensors, ensuring the quality and safety of consumables [240,241]. Industrial process monitoring can leverage optical biosensors for precise and continuous monitoring of parameters crucial for manufacturing processes, enhancing efficiency and product quality [242,243]. In veterinary medicine and agriculture, optical biosensors offer opportunities for disease detection in livestock, monitoring environmental parameters, and optimizing agricultural practices for sustainable food production [244,245]. Disease diagnosis and healthcare stand at the forefront of optical biosensor applications, enabling the early detection of diseases, including cancer and infectious diseases, and thereby facilitating timely intervention and improved patient outcomes [246,247]. Biotechnology and biosciences will see advancements in genetic analysis, protein detection, biomolecular interactions, resulting in innovation in research and development [248]. The military and defense sector can utilize optical biosensors for the rapid and sensitive detection of chemical and biological threats, enhancing national security [249]. Environmental inspection and safety benefits are the ability of optical biosensors to detect pollutants, pathogens, and hazardous substances, aiding in environmental monitoring and protection [250,251]. Considering the COVID-19 pandemic, optical biosensors have emerged as critical tools for the rapid detection of the virus, contributing to disease surveillance, outbreak control, and public health efforts [252,253,254]. As the field continues to evolve, optical biosensors hold tremendous promise in addressing diverse challenges across various sectors, ushering in a new era of sensing technology. Thus, optical biosensors have emerged as transformative tools in cancer diagnostics due to their high sensitivity, label-free detection capabilities, and potential for miniaturization and real-time monitoring. Among the sensors discussed in this article, SPR sensors, fluorescence-based sensors, Raman spectroscopy, and optical fiber-based sensors have shown remarkable promise in the early detection and classification of various cancer types. These sensors can detect several cancer biomarkers, circulating tumor cells (CTCs), exosomes, and other cancer-associated molecules at very low concentrations, offering a significant advantage over traditional diagnostic methods. For example, PCF SPR biosensors have demonstrated high resolution and selectivity in detecting breast cancer cell lines such as MDA-MB-231 and MCF-7. Despite these advancements, in optical sensing techniques, several challenges persist in detecting the cancer. Some of the prominent challenges are listed as follows.

- The biocompatibility and stability of sensing materials in complex biological environments are a major limitation for the sensing devices used today [255].
- Production and standardization of sensor fabrication, along with the stable performance across different platforms, is a major challenge [255].
- A fluctuating signal-to-noise ratio in complex blood or tissue extracts can affect sensor sensitivity [256].
- The integration of these sensors with clinical workflows and real-time data interpretation tools, particularly in point-of-care settings, is still at an early stage and needs further development [257].

To overcome these challenges, several strategies can be adopted, such as the following.

- Development of novel plasmonic and 2D materials like graphene, MXenes, and MoS_2_ which have the potential to significantly enhance sensor sensitivity and stability [258].
- The merger of machine learning (ML) and artificial intelligence (AI) algorithms for signal pattern recognition and classification of cancer types from the optical sensor outputs [259].
- Developing microfluidics-based lab-on-a-chip systems to ensure sample handling accurately and sensor integration in portable, automated platforms [260].

Lastly, optical sensors offer a powerful and non-invasive platform for cancer diagnostics. With continued innovation in materials science, data analytics, system integration, and using AI-based techniques. These devices can play a critical role in the future of personalized and precision oncology. Finally, in Table 6, we present the monetary aspects of using these biosensors in cancer diagnosis and detection.

## 5. Conclusions

This investigation has provided a comprehensive overview of various optical biosensors employed for the detection of cancer cells, related biomarkers, and different related prospects. The discussed technologies present a wide range of approaches, including SPR sensors, LSPR sensors, colorimetric sensors, fluorescence-based sensors, photonics and waveguide sensors, Raman spectroscopy-based sensors, and fiber optic sensors. Each of these technologies is explained in an exhaustive manner, offering unique advantages in terms of sensitivity, specificity, cost-effectiveness, and suitability for cancer detection. These optical biosensors hold significant promise in advancing cancer diagnostics by enabling early detection and improving patient health. Moving forward, continued research and development in this field will further enhance the capabilities and applicability of optical biosensors in the fight against cancer.

## Figures and Tables

**Table 1:** Methodologies for cancer detection.

Methodologies	Forms of Cancers	Advantages	Limitations	Ref
Biopsy	Breast, prostate cancer	High accuracy, provides definitive diagnosis	Invasive, painful, time-consuming, risk of infection, not suitable for early detection without visible lesions	[25,26]
Magnetic resonance imaging (MRI)	Gastro intestine, brain, prostate cancer	Non-invasive, provides high-resolution images, effective for soft tissue examination	Expensive, time-intensive, limited availability, may require contrast agents, not always specific to cancer	[27,28]
Ultrasound Imaging	Breast, and prostate cancer	Non-invasive, real-time imaging, widely available, relatively low cost	Limited resolution, operator-dependent, less effective for detecting small or deep tumors	[29,30]
X-ray	Lung, colorectal cancer	Quick, non-invasive, cost-effective, widely available	Exposure to ionizing radiation, limited sensitivity for small or early-stage cancers	[31,32]
Computed tomography (CT) scans	Breast, lung, and prostate cancer	High-resolution, provides 3D imaging, effective for staging and monitoring tumors	High radiation dose, expensive, limited effectiveness in detecting small or early-stage cancers	[33,34]
Endoscopy	Oral, gastric, and colon cancer	Allows direct visualization of tissues, can collect biopsy samples during the procedure	Invasive, uncomfortable for patients, requires sedation, limited to accessible regions	[35,36]
Occult blood detection	Colon cancer	Non-invasive, cost-effective, simple screening tool	Low specificity, prone to false positives and false negatives, requires follow-up tests for confirmation	[37,38]
Prostate-specific antigen level detection	Prostate cancer	Non-invasive, widely used screening tool, helpful for monitoring progression	Low specificity, risk of overdiagnosis, elevated levels may result from non-cancerous conditions	[39,40]
Mammography	Breast cancer	Non-invasive, effective for early detection, widely used	Risk of false positives and false negatives, involves exposure to low-dose radiation, less effective for dense breast tissue	[41,42]
Papanicolaou test	Cervical cancer	Non-invasive, cost-effective, widely used for early detection	Requires regular follow-up, subjective interpretation, less effective for adenocarcinomas	[43,44]

**Table 2:** Cancer biomarkers reported via various calorimetric paper-based sensors.

Target	PAD	Element	Detection System	Range	LOD	Ref
Protein CA−125	SPOT test	Ab	AuNPs+Ag enhancements	30 to 00 U/mL	30 U/mL	[154]
	SPOT test	Ab	Cys−AuNPs	0.1 to 100 U/mL	0.413 U/mL	[155]
	SPOT test	Ab	Cys−AuNPs	0.1 to 100 ng/mL	1.054 ng/mL	[155]
Protein CEA	µPAD	Ab	HRP+TMB	0.5 to 70 ng/mL	0.015 ng/mL	[156]
	µPAD	Ab	HRP+TMB	0 to 40 ng/mL	2 ng/mL	[157]
	SPOT test	Ab	nanocompositeperoxidase−like activity+TMB	0.002 to 75 ng/mL	0.51 pg/mL	[158]
Protein PSA	µPAD	Ab	AuNPs	0.5 to 50 µg/L	≤360.2 ng/L	[159]
	µPAD	Ab	${Fe}_{3}O_{4}\;\mathrm{to}\;PB$	3 to 80 ng/mL	2.7 ng/mL	[160]
Enzymes ALPPEAK1	µPAD	Ab	PNPP	30 to 500 U/mL	−−	[161]
	SPOTtest	Ab	AuNPs+HNB	$1\times 10^{-9}\;g/mL–\;1\times 10^{-5}\;g/mL$	1.0 ng/mL	[162]
Growth Factor HER2	LFA	aptamer	AuNPs+biotin+streptavidin	0 to 50 nM	20 nM	[163]
Nucleic acid µRNA155	LFA	ssDNA	AuNPs+biotin+streptavidin	0.01 to 5 nM	0.061 nM	[164]
Nucleic acid µRNA210	LFA	ssDNA	AuNPs+biotin+streptavidin	0.05 to 10 nM	0.085 nM	[156]
Nucleic acid µRNA215	LFA	ssDNA	AuNPs+biotin	0.075 to 0 nM	60 pM	[165]

**Table 3:** Nanoparticles exhibiting colorimetric behavior through *LSPR.*

Nanoparticles	Description	Ref
$TiO_{2}/SnOx-Au$	Immunoassay sensor used for tumor marker	[157]
NiO	Breast cancer detection	[158]
Au	Spermidine and spermine detection	[159]
Cu	DNA detection at femtomolar level	[160]
Pt	Hg ions in water	[161]
$Au-{Cu}_{9}S_{5}$	Photothermal therapy in the second NIR window	[162]
Au/Ag	Glucose sensing	[163]
${Bi}_{2}{Se}_{3}-Au$	Cancer biomarker detection	[164]

**Table 4:** Biomarker detection in cancerous cells using *FRET* technology.

Types	Biomarker	C*	D*	A*	DL	Ref
AH	C−MYC	Breast	Cy3	Cy5	0.6 pM	[176]
AH	µRNAs	Lung	QD	Cy5	0.54 fM	[177]
T*	TK1 mRNA	Liver	Cy3	Cy5	0.33 nM	[178]
AH	µRNAs	Cervical	BHQ2	BHQ2	0.8 pM	[179]
MB	TK1 mRNA	Liver	TAMRA	TAMRA	2.0 nM	[180]
MB	µRNAs	Breast	ROX	ROX	0.18 pM	[181]
AH	µRNAs	Breast	BHQ2	BHQ2	0.74 fM	[182]
MB	µRNA	Cervical	Cy5	AuNPs	0.9 nM	[183]
AH	µRNAs	Cervical	Cy5	Cy5	2.14 pM	[184]
T*	Survivin Mrna	Breast	Cy5	Cy5	1.5 nM	[185]

T* = Tetrahedral, C* = cancer, D* = Donar, A* = acceptor, DL = detection limit.

**Table 5:** Cancer analysis using body fluids adopted for *SERS.*

Body Fluid	Cancer	SERS Platform	Methods	Ref
Urine 1	Prostate/pancreatic	3D−PCN	RNN, CNN	[216]
Urine 2	Prostate/pancreatic	3D stack Ag NWs GFF SERS	PCA, OPLS−DA	[217]
Serum 1	Lung	Au NSs	PCA RCKNCN	[218]
Serum 2	Sjogren syndrome diabetic nephropathy	${Ag}_{2}O-Ag-PSB$	SVM	[219]
Serum 3	Bladder/adrenal	AgNPs	1D−CNNGRAD−CAM	[220]
Serum Exosomes	Breast cancer	Au Sts	MCR−ALS, PCA−LDA	[221]
Secretomes	Cervical	Drop casting AgNPs	PCA, SVM	[222]
HER2 cells	Breast cancer	Au Sts	ANN	[223]

**Table 6:** Comparison of the cost-effectiveness of various sensor types.

Type of Sensor	Production Cost	Remark on Cost-Effectiveness	Ref
SPR sensors	Moderate to high	High fabrication cost due use of noble material coatings like Au, Ag, Pt, and require complex optical alignment	[261]
LSPR sensors	Low to moderate	Lower cost than SPR because of simpler setups and nanostructure-based signal enhancement	[262]
Colorimetric sensors	Low	Simple fabrication process using paper-based substrates, and no external instrumentation needed	[263]
Fluorescence-based sensors	Moderate	Requires labeling and excitation sources sometimes can be often limited by photobleaching	[264]
Photonics and waveguide sensors	High	Due to the need to fabricate photonic chips and requirement of cleanroom facilities	[265]
Raman spectroscopy sensors	High	Due to the need for high-power lasers, spectrometers, and filters	[266]
Fiber-optic sensors	Moderate	Mostly depends on fiber type, coatings, and interrogation systems	[267]

## Data Availability

The raw data supporting the conclusions of this article will be made available by the authors upon request.

## Abbreviations

Abs	Antibodies
AFM	Atomic-force microscopy
Ag	Silver
Al	Aluminum
APTES	Aminopropyl-triethoxysilane
AS	Amplitude sensitivity
AS 1411	Nucleole aptamers
Au	Gold
AuNS	Gold nanostar
BCP	Bromocresol purple
BSA	Bovine serum albumin
CK17	Cytokeratin 17
CVD	Chemical vapor deposition
CT	Computed tomography
DI	De-ionized
DMMP	Dimethyl-methyl-phosphonate
EMD	External metal deposition
EXO	Exosomes
FA	Folic acid
FEM	Finite element method
FPI	Fabry-Perot interferometer
FRET	Fluorescence resonance energy transfer
GC	Gas chromatography
GNRs	Gold nanorods
GOD	Glucose oxidase
IMD	Internal metal deposition
ITO	Indium tin oxide
LFAs	Lateral flow assays
LFS	Lateral flow strip
LSPR	Localized surface plasmon resonance
mb-RCA	Multi-branched rolling circle amplification
MgF2	Magnesium fluoride
MEMS	Micro-electromechanical systems
MIO	Microporous inverse opal
MMV	Micromechanical valves
MRI	Magnetic resonance imaging
NOA-81	Norland optical adhesive
NIR	Near-infrared
NPs	Nanoparticles
OF	Optical fiber
PBS	Phosphate buffer saline
PCW	Photonic crystal waveguide
PCFs	Photonic crystal fibers
PDMS	Polydimethylsiloxane
PEG-400	Polyethylene glycol
POC	Point of care
POCT	Point-of-care testing
PTT	Photothermal therapy
PUA	Polyurethane acrylate
RI	Refractive index
RW	Resonant wavelength
Si	Silicon
SEM	Scanning electron microscopy
SERS	Surface-enhanced Raman spectroscopy
SPs	Surface plasmons
SPP	Surface plasmon polariton
SPR	Surface plasmon resonance
SPW	Surface plasmon wave
SR	Sensor resolution
TCOs	Transparent conductive oxides
TEM	Transmission electron microscopy
TE	Transverse electric
TiO_2_	Titanium dioxide
TIR	Total internal reflection
TM	Transverse magnetic
TMDCs	Transition metal dichalcogenides
VIS-NIR	Visible-near-infrared
VOCs	Volatile organic compounds
WS	Wavelength sensitivity
2D	Two-dimensional
3D	Three-dimensional
